# The apical protein Apnoia interacts with Crumbs to regulate tracheal growth and inflation

**DOI:** 10.1371/journal.pgen.1007852

**Published:** 2019-01-15

**Authors:** Kassiani Skouloudaki, Dimitrios K. Papadopoulos, Pavel Tomancak, Elisabeth Knust

**Affiliations:** Max-Planck Institute for Molecular Cell Biology and Genetics, Dresden, Germany; University of California Santa Barbara, UNITED STATES

## Abstract

Most organs of multicellular organisms are built from epithelial tubes. To exert their functions, tubes rely on apico-basal polarity, on junctions, which form a barrier to separate the inside from the outside, and on a proper lumen, required for gas or liquid transport. Here we identify *apnoia* (*apn*), a novel *Drosophila* gene required for tracheal tube elongation and lumen stability at larval stages. Larvae lacking Apn show abnormal tracheal inflation and twisted airway tubes, but no obvious defects in early steps of tracheal maturation. *apn* encodes a transmembrane protein, primarily expressed in the tracheae, which exerts its function by controlling the localization of Crumbs (Crb), an evolutionarily conserved apical determinant. Apn physically interacts with Crb to control its localization and maintenance at the apical membrane of developing airways. In *apn* mutant tracheal cells, Crb fails to localize apically and is trapped in retromer-positive vesicles. Consistent with the role of Crb in apical membrane growth, RNAi-mediated knockdown of Crb results in decreased apical surface growth of tracheal cells and impaired axial elongation of the dorsal trunk. We conclude that Apn is a novel regulator of tracheal tube expansion in larval tracheae, the function of which is mediated by Crb.

## Author summary

Tubular organs, such as the fruitfly airways, comprise essential functional pipes through which gas and liquid are transported. They consist of highly polarized epithelial cells that form a barrier between air and the larval body. However, during larval development, these tubes, though very rigid due to the presence of cuticle, need to rapidly grow in size within short time-windows. To control their growth, cells have to regulate the apical membrane surface of the epithelial cells. In this work we have discovered a new gene called *apnoia*, which is important for tracheal growth and inflation at larval stages. We demonstrate that Apnoia protein is localized in the apical membrane of tracheal epithelia cells, where it is required for apical membrane expansion. This function is mediated by regulating the proper localization and maintenance of the well-known apical determinant Crumbs. Both Apnoia and Crumbs proteins are required for expansion of the apical cell surface and, thereby, tube elongation. Such a mechanism is required to support the complex morphogenetic events that the tracheal system undergoes during development.

## Introduction

Animal organs consist of epithelial tissues, which form the boundaries between internal and external environment [1–3]. During development, epithelia are instrumental to shape the various organs. Many epithelial tissues form tubular organs, such as the gut, the kidney or the respiratory system. A fundamental feature of epithelial tubes and sheets is to keep the balance between the maintenance of structural integrity and tissue rigidity during organ growth and morphogenesis. To understand how this balance is achieved during rapid, temporally regulated developmental transitions from juvenile to adult body shapes, several studies in various animal models have focused on elucidating how cell proliferation, cell polarity, cell shape changes and trafficking contribute to the formation of the tubular lumen length and diameter [4–7]. The correct coordination of these processes is crucial for normal organ function. This is reflected in the fact that several human diseases are linked to defects in epithelial tube formation and maintenance, such as polycystic kidney disease or cystic fibrosis [8–11].

The developing tracheae of *Drosophila melanogaster*, a network of branched epithelial tubes that ensure oxygen supply to the cells of the body, has emerged as an ideal system to study cell fate determination and morphogenesis of epithelial tubes. The available genetic tools, as well as the ease to image the tracheal system in the fly embryo, has provided detailed insights into the developmental processes required to form tubular structures with defined functional lumens and have contributed to elucidating the interplay between tissue growth, differentiation and cell polarity [12–15].

The stereotypically branched tracheal system of *Drosophila* is set up at mid-embryogenesis. Once a continuous tubular network has formed, the tube expands to accommodate an increased oxygen supply to all tissues during animal growth. Tube expansion occurs by growth along the diameter and along the anterior-posterior axis. Growth is accompanied by the formation of a transient cable, comprised of a chitinous apical extracellular matrix (aECM), which fills the lumen of the tube. The generation of this cable requires the secretion of chitin and chitin-modifying enzymes. Mutations in genes affecting secretion or organization of the chitin cable result in excessively elongated tracheal tubes or tubes with irregular diameter (with constricted and swollen areas along the tube) [15–17]. Axial growth, on the other hand, depends on the proper elongation of tracheal cells along the anterior-posterior axis. At later stages of embryogenesis, the lumen becomes cleared and filled with air.

After hatching, the larvae undergo two molts, a process during which animals rapidly shed and replace their exoskeleton with a new one, bigger in size. For this, a new chitinous aECM is secreted apically, thus surrounding the old tube. Remodeling of this aECM permits tissue growth between larval molts. The molting process is initiated by the separation of the old aECM from the apical surface of the epithelial cells and the secretion of chitinases and proteinases, which partially degrade the old cuticle. The remnants of the old cuticle in each metamer are shed through the spiracular branches. This process, called ecdysis, is followed immediately by clearance of the molting fluid and air filling [18–20]. Interestingly, while the diameter of the dorsal trunk only increases at each molt, tube length increases continuously throughout larval life, particularly during intermolt periods [14]. Despite the importance of tube expansion and elongation for larval development [18], the underlying mechanisms that control tracheal growth at this stage remain poorly understood.

A well-established regulator of apical domain size in developing epithelia is Crumbs (Crb). Crb is a type I transmembrane protein, which acts as an apical determinant of epithelial tissues [21]. It has a large extracellular, a single transmembrane and a short cytoplasmic domain. Loss- and gain-of-function experiments have shown that apical levels of Crb are important for proper cell polarity, tissue integrity and growth. For instance, absence of Crb in embryonic epithelia results in loss of apical identity and disruption of epithelial organization [22–24]. In contrast, overexpression of Crb triggers apical membrane expansion, which leads to a disordered epithelium, abnormal expansion of tracheal tubes and/or tissue overgrowth [21,25–33]. These results underscore the importance of Crb levels for epithelial development and homeostasis.

Several mechanisms have been uncovered that ensure proper levels of apical Crb. These include: stabilization of Crb at the membrane, mediated through interactions of its cytoplasmic domain with scaffolding proteins, e.g. Stardust (Sdt), or by homophilic interactions between Crb extracellular domains [26,34–36], regulation of Crb trafficking, including endocytosis by AP-2, Rab5 or Avalanche, membrane delivery by Rab11, recycling by the retromer and endocytic sorting by the ESCRT III component Shrub/Vps32 [29,33,37–41].

To gain further insight into the molecular mechanisms that regulate Crb and its activity during epithelial growth, we set out to identify novel interacting partners of Crb using the yeast two-hybrid system. One of the candidates identified, CG15887, encodes a transmembrane protein, which localizes to the apical surface of tracheal tubes. We found that the CG15887 protein physically interacts with Crb. Based on the phenotype of mutations in CG15887, which is characterized by defects in tracheal growth and inflation during larval stages, we named this gene *apnoia* (*apn*). *apn* mutant animals die as second instar larvae with dorsal trunks displaying reduced axial growth and impaired apical surface area expansion, resulting in shorter tubes. This phenotype is correlated with the absence of Crb from the apical surface. RNAi knock-down of *crb* phenocopies the *apn* mutant phenotype of impaired longitudinal growth. These results identify Apn as a novel regulator of tracheal tube growth in the larvae, which acts through Crb to control axial tube expansion.

## Results

### Apnoia is an apical transmembrane protein expressed in *Drosophila* tracheae

To identify novel interactors of Crb, we searched for binding partners using a modified yeast two-hybrid screen (MBmate Y2H) [42,43], allowing bait and prey to interact at the yeast plasma membrane. The bait consisted of the C-terminal-most extracellular EGF (epidermal growth factor)-like repeat, the transmembrane domain and the cytoplasmic tail of *Drosophila* Crb. One of the Crb interacting clones contained a 414bp cDNA insert encoded by the full-length CG15887 gene. Based on the tracheal inflation phenotype described below we named the gene *apnoia* (*apn*) (άπνοια, Greek for: lack of air).

The *apn mRNA* encodes a single protein isoform of 137 amino acids. Apn is predicted to contain a signal peptide at the amino terminus (1–23 aa) and two transmembrane domains (amino acids 50–72 and 79–101), based on the TMHMM transmembrane algorithm prediction [44]. Both the amino and carboxy terminus are located extracellularly, separated by a small intracellular loop (S1 Fig). The PFAM algorithm (PFAM domains database 27.0) predicts that Apn contains two LPAM domains (47–56 aa and 78–90 aa), known as prokaryotic membrane lipoprotein lipid attachment sites. Apn is highly conserved within the insect order (S1 Fig) but does not appear to have a true orthologue in vertebrates.

**Fig 1:**
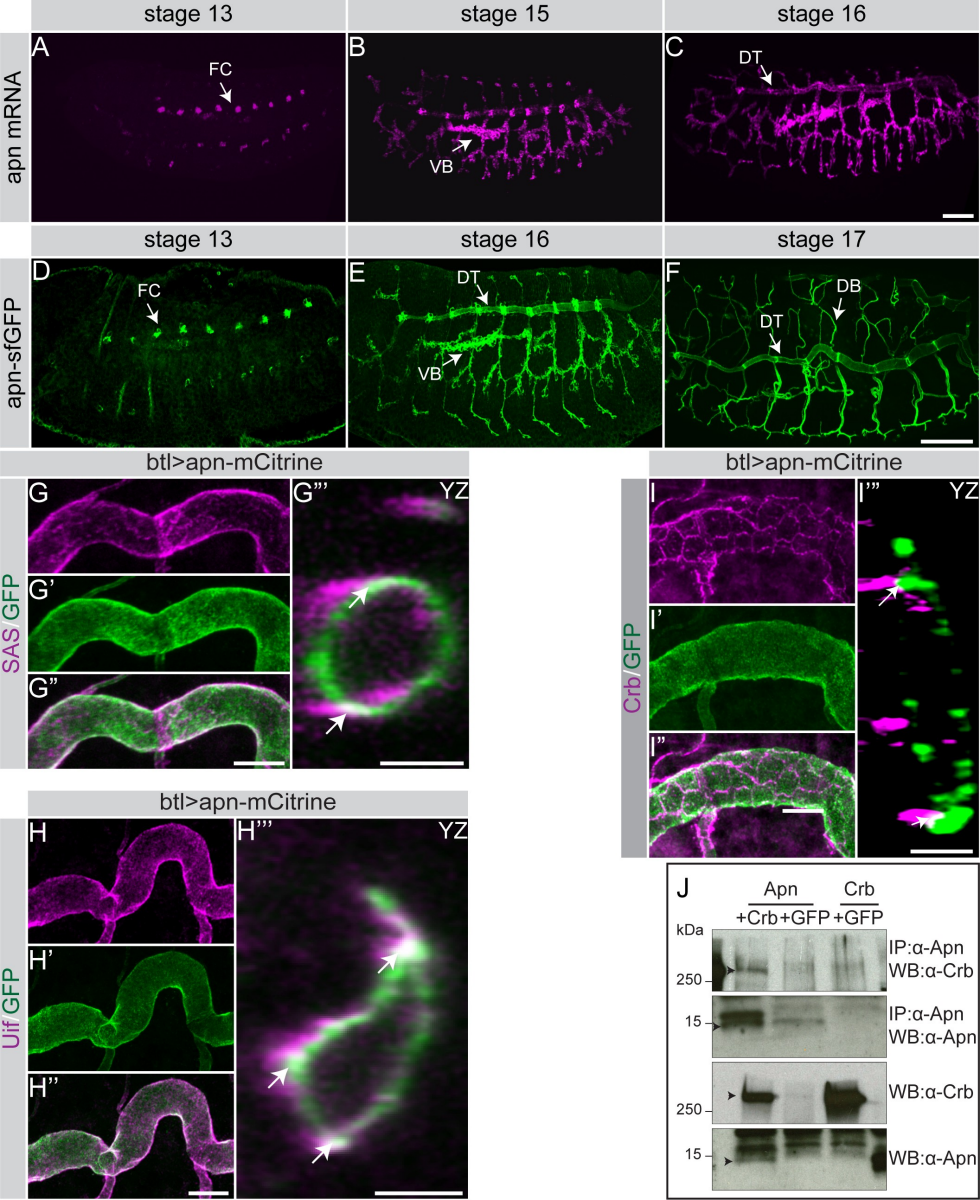
Apn is a small transmembrane protein detected on the apical membrane of the tracheal epithelium. (**A-F**) Expression of *apn* as detected by whole mount *in situ* hybridization (A-C) and *fosapn*_*sfGFP*_ expression (D-F) in lateral views of stage 13, 15, 16, 17 embryos. *apn* RNA and protein appear first in fusion cells (FC) and later in the dorsal trunk (DT), visceral branches (VB) and dorsal branches (DB). Scale bar: 50μm. (**G-H‴**) Apn-mCitrine (green) localizes on the apical plasma membrane together with the apical markers SAS (G, G’, G”) and Uif (H, H’, H”), as revealed by z-projections of stage 17 tracheal tubes and optical cross sections (G‴ and H‴). Scale bars: (G-G” and H-H”) 10μm, (G‴) 5μm, (H‴) 5μm. Arrows in G‴ and H‴ point to protein overlap. **(I-I‴)** Tracheae of btl>apn-mCitrine stage 17 embryos stained with anti-Crb (magenta) and anti-GFP (green). Cross section indicates the apical localization of Apn as compared to subapical localization of Crb. Arrows in I‴ point to protein overlap. Scale bars: (I-I”) 10μm, (I‴) 3μm. **(J)** Co-immunoprecipitation experiments from S2R^+^ cells expressing the following constructs: UAS-Crb^FL^+UAS-Apn^FL^ or UAS-Apn^FL^+UAS-GFP or UAS-Crb^FL^+UAS-GFP. Crb is co-immunoprecipitated with Apn, but not with GFP (used as negative control).

To determine the tissue distribution and subcellular localization of *apn* mRNA and Apn protein we performed *in situ* hybridizations and immunostainings of wild-type or transgenic animals, which either carried the *fosapn*_*sfGFP*_, a fosmid encoding the Apn protein C-terminally tagged with superfolded (sf) GFP [45], or a UAS-transgene encoding fluorescently-tagged Apn (UAS-*apn*_mCitrine_). In addition, anti-Apn antibodies were raised in rabbits against a peptide of the N-terminal extracellular domain (aa 24–40). Expression of both *apn* mRNA (Fig 1A–1C) and Apn protein (Fig 1D–1F and S2A and S2B Fig) were first detected in embryos at stage 13 in tracheal fusion cells. During embryonic stages 15 and 16, expression could also be detected in the dorsal and lateral trunks, in the visceral and dorsal branches and in the transverse connective branches. In the larvae, Apn is continuously expressed in the entire tracheal system (S2C and S2D Fig). As shown by antibody staining or Apn_mCitrine_ fluorescence, Apn is restricted to the apical plasma membrane, where it co-localizes with the apical markers Stranded at second (Sas) (Fig 1G–1G” and cross section in 1G‴) and Uninflatable (Uif) (Fig 1H–1H” and cross section in 1H‴). Apn co-localizes with Crb in the subapical region, a small region of the apical membrane apical to the adherens junctions (AJ) (Fig 1I–1I” and cross section in 1I‴).

**Fig 2:**
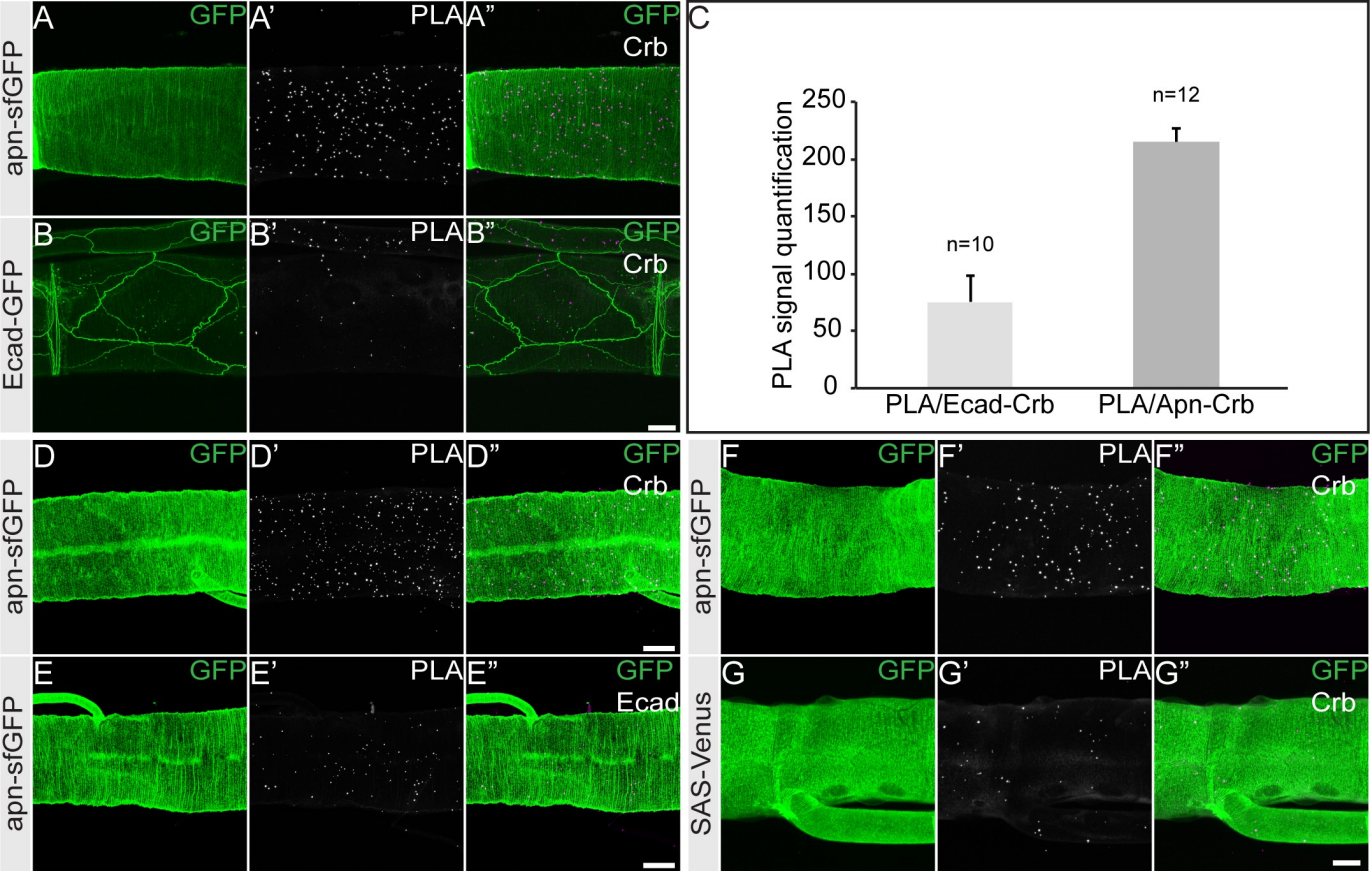
Apnoia interacts with Crb. **(A-B”)** Proximity ligation assay (PLA) shows a robust increase in number of interactions between Crb and Apn (spots in magenta) in fos*apn*_sfGFP_ larval tracheae (A-A”) as compared to control tracheae (*D*Ecad-GFP) (B-B”). Scale bar: 20μm. **(C)** Quantification of the PLA signal produced between Crb and Apn as compared to Crb and *D*Ecad. **(D-E”)** Proximity ligation assay (PLA) shows increased numbers of interactions between Crb and Apn (spots in magenta) in fos*apn*_sfGFP_ larval tracheae (D-D”) when Crb and GFP antibodies were used as compared to fos*apn*_sfGFP_ larval tracheae (E-E”) when *D*Ecad and GFP antibodies were used. Scale bar: 50μm. **(F-G”)** Proximity ligation assay (PLA) shows a robust increase in number of interactions between Crb and Apn (spots in magenta) in fos*apn*_sfGFP_ larval tracheae (F-F”) as compared to a control apical protein *SAS*-Venus (G-G”). Scale bar: 50μm.

This co-localization and the interaction in the yeast 2-hybrid system (S2E Fig) prompted us to further analyze the interaction between Apn and Crb in co-immunoprecipitation experiments. Full-length Apn (Apn^FL^) expressed in S2R^+^ cells co-immunoprecipitated the full-length Crb (Crb^FL^) (Fig 1J). *In situ* interactions between Crb and Apn were corroborated by Proximity Ligation Assays (PLA) [46] using *fosapn*_*sfGFP*_. We found that Crb and Apn-sfGFP interact in the larval tracheae (Fig 2A–2A”, 2D–2D” and 2C), whereas no interaction between Apn and the *Drosophila* E-cadherin, tagged to GFP (*D*Ecad-GFP) (negative control), was detected (Fig 2B–2B”, 2E–2E” and 2C), indicating that the observed signal was specific for the Crb-Apn interaction. To exclude any random interactions between Crb and Apn in the apical domain we have tested a different apical protein (SAS-Venus)[47] for its interaction with Apn and found no increased PLA signal as compared to the signal between Crb and Apn (Fig 2F–2F” and 2G–2G”).

### *apnoia* is required for tracheal tube growth

To address possible functions of *apn* in tracheal development, we generated a knockout line by CRISPR-Cas9, in which the open reading frame of *apn* was replaced by DsRed (*apn*^*1*^). No Apn protein could be detected with the anti-Apn antibody in homozygous *apn*^*1*^ mutant larvae and embryos (S2B, S2D and S2F Fig). In addition, no interaction between Crb and Apn was detected in *apn*^*1*^ mutant tracheae in PLA assays as compared to wild type tracheae (S2G–S2G’ Fig).

**Fig 3:**
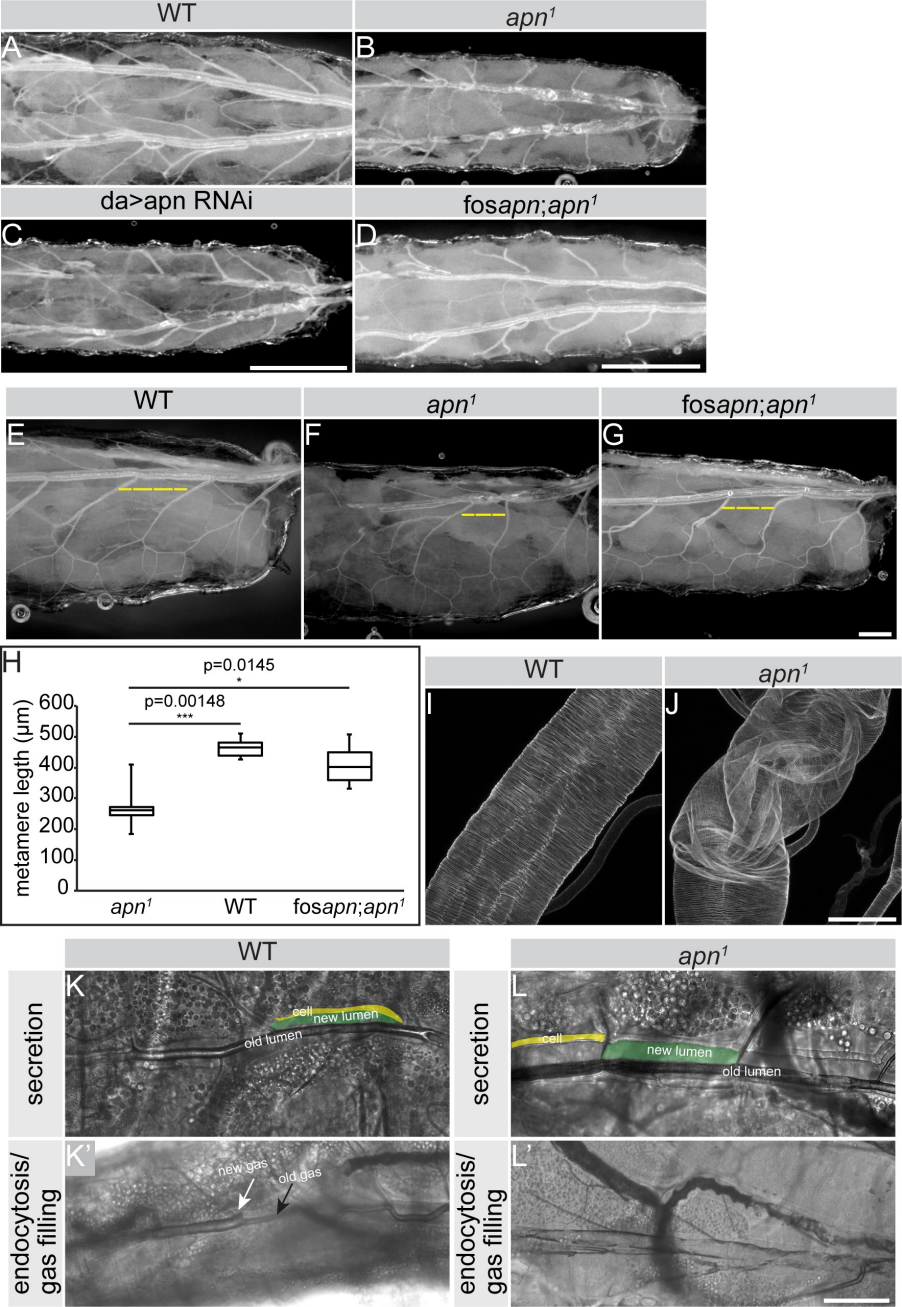
Apn is essential for tracheal tube maturation. (**A-D**) Brightfield images showing the tracheal tubes of second instar larvae. Rigid and gas filled tracheal tubes are present in wild type (WT) larvae (A), whereas absence of *apn* causes twisted and gas deficient tubes (B). *apn* down-regulation (*apn* RNAi) recapitulates *apn*^*1*^ mutant tracheal defects (C). Tube morphology defects are rescued by one genomic copy of *apn* (fos*apn*_mCherry.NLS_) (D). Anterior is to the left. Scale bar: 500μm. (**E-G**) The 9^th^ metamer of the dorsal trunk (yellow dotted line) of *apn*^*1*^ mutant second instar larvae (F) is shorter than that of WT larvae (E). Expression of one genomic copy of *apn* (fos*apn*_mCherry.NLS_) significantly rescues the metamer elongation defects of *apn* mutant larvae (G). Anterior is to the left. Scale bar: 200μm. **(H)** Quantification of the length of the 9^th^ metamer of second instar tracheal tubes of WT, *apn*^*1*^ mutants and *apn*^*1*^ mutants rescued with one extra *apn* copy (fos*apn*_mCherry.NLS_;*apn*^*1*^). Measurements were pooled from 6 larvae. (**I-J**) Chitin binding probe (CBP) allows the visualization of the tracheal tube structure. Note the wrinkled and twisted tube of *apn*^*1*^ mutants (J) as compared to that of WT (I) larvae. Scale bar: 50μm. (**K-L’**) Brightfield images to show dorsal trunk diameter expansion (K, L) and gas filling (K’, L’) of the newly formed tracheal tube of WT (K, K’) and *apn*^*1*^ mutant (L, L’) second larval instar. Yellow indicates the tracheal cells that line the newly formed lumen (green). Note the bubble filling the newly formed lumen in WT (white arrow) (K’), which is absent in the tube of *apn*^*1*^ mutants (L’), which fails to fill with gas. Scale bar: 50μm.

*apn*^*1*^ mutant tracheae displayed wild type morphology in all embryonic stages, even in embryos derived from *apn*^*1*^ mutant germ line clones (S2H Fig). However, *apn*^*1*^ mutant larvae died at second instar with reduced body size and unusually twisted and uninflated tracheal tubes (compare Fig 3A and 3B, S2I and S2K Fig). The phenotype is mostly manifested in the posterior tracheal metameres 9 (Tr9) and 10 (Tr10). Similar phenotypes were observed in larvae that carry *apn*^*1*^ in trans to Df(3R)Exel8158 (S2J Fig), a chromosomal deletion that includes the *apn* locus, as well as in larvae upon knock-down of *apn* by RNAi in the tracheae (Fig 3C and S2K, S3A and S3B Figs). In addition, the length of the dorsal trunk was significantly reduced, as revealed by measurements of the posterior metamer length (Fig 3E, 3F and 3H). The morphological and growth defects were rescued by one copy of a fosmid containing the complete *apn* locus (fos*apn*_mCherry.NLS_) (Fig 3D, 3G and 3H), whereas a cDNA of Apn expressed in the tracheae rescued the phenotype in only 20% of the larvae (compare S3A, S3D, S3C and S3F Fig).

The uninflated tubes observed in *apn*^*1*^ deficient animals suggested defects in tracheal maturation. In wild-type embryos as well as in each molting step of larval development, tracheal maturation is characterized by distinct sequential processes: i) secretion of a chitinous apical extracellular matrix (aECM) into the lumen, which confers rigidity to the tube and is responsible for tube expansion; ii) a pulse of endocytosis, resulting in the removal of luminal proteins, and iii) liquid clearance and air filling [16,48]. Electron micrographs of *apn*^*1*^ mutant larvae revealed a disorganized lumen with “tongues” of cellular protrusions into the lumen (S3G and S3H Fig). This phenotype is probably a consequence of the irregularly twisted tubes (compare Fig 3I and 3J) and not due to defects in cuticle organization, since the two different cuticular layers, epicuticle and procuticle, were normally formed and the spaced thickenings formed by the aECM (taenidia) [49] appeared similar to that of wild type tubes (Fig 3I and 3J and S3G’ and S3H’ Fig). This conclusion is further supported by the normal expression of Dumpy (Dp) and Piopio (Pio), two zona pellucida (ZP) domain proteins secreted into the lumen [50,51] (S3I–S3J’ Fig). The second maturation step, consisting of the endocytosis of luminal proteins, was neither impaired in *apn*^*1*^ mutant tubes. Using the heterologous secreted mCherry-tagged protein ANF (UAS-ANF-mCherry, a rat Atrial Natriuretic Factor) [52] revealed normal secretion and endocytosis in tracheal cells deficient for Apn (compare S3K–S3L’ Fig). However, the last maturation steps, involving the liquid clearance and gas filling, were strongly affected in *apn*^*1*^ mutant tracheae (compare Fig 3K, 3K’, 3L and 3L’). We could exclude leakage of the septate junctions (SJ) and hence loss of paracellular barrier from being a cause of this phenotype, since Contactin (Con) and Discs Large (Dlg), two SJ components [53,54] were properly localized in the tracheae of *apn*^*1*^ mutants (S3M–S3N’ Fig).

Taken together, our data demonstrate that loss of *apn* affects the late steps of tracheal tube maturation, including liquid clearance and gas filling, and impairs the growth and morphology of the dorsal trunk at the second larval stage.

### Apn supports apical membrane growth in larval tracheae

**Fig 4:**
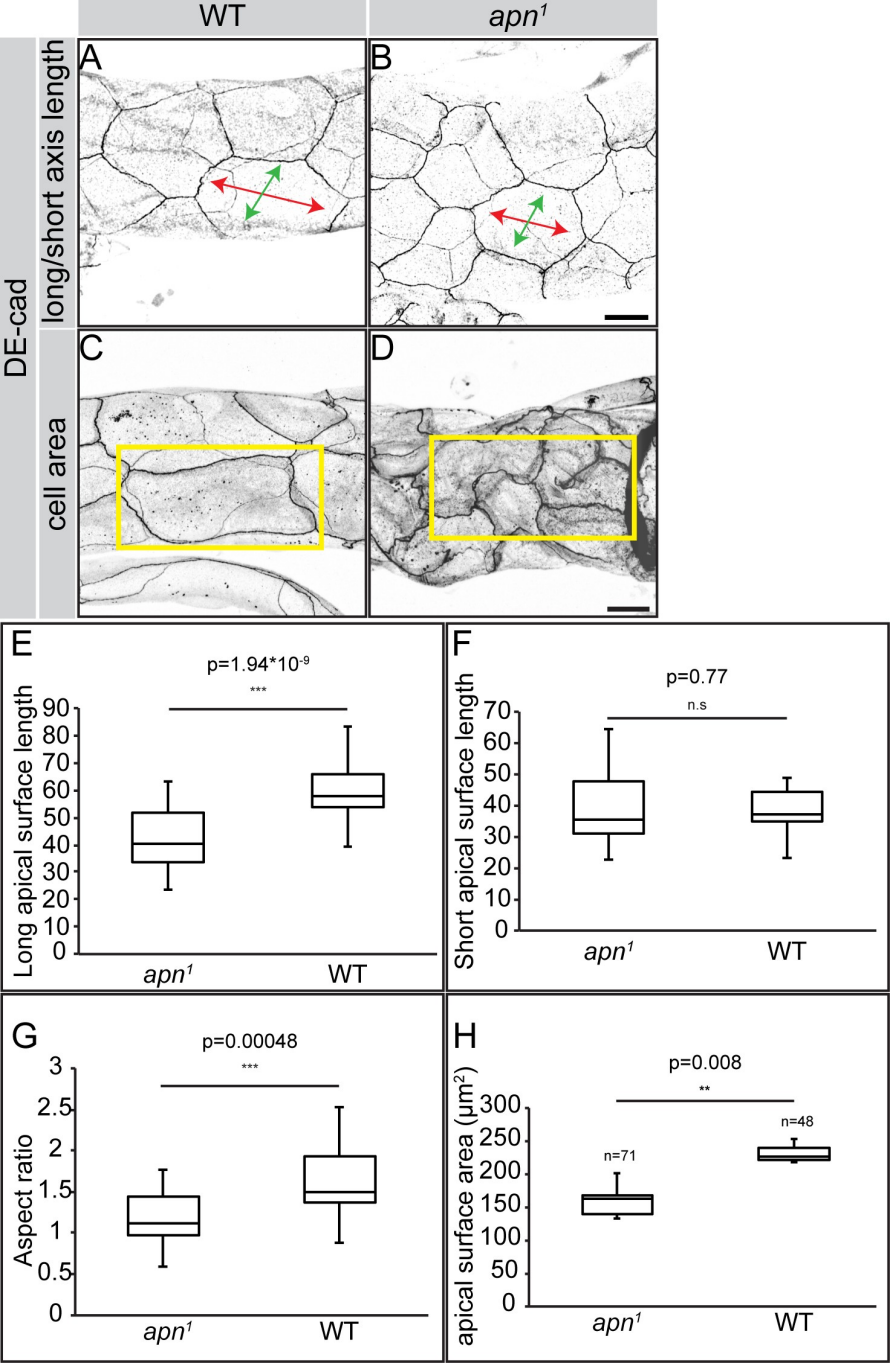
Apn is required for longitudinal elongation of tracheal cells. (**A-D**) Tracheal tubes of wild type (WT; A, C) and *apn*^*1*^ mutant (B, D) second instar larvae stained with anti-*D*Ecad. The long and short axes of the apical surfaces (A, B) are indicated by red and green arrows, respectively, and the cells by yellow rectangles (C, D). Note that the number of cells occupying identical areas in the tracheal tube is higher (~ 4 cells) in *apn*^*1*^ mutants (D) than in WT (~1 cell) (C). Scale bars: 20μm. (**E-H**) The median length of the long (E) axis is significantly smaller in *apn*^*1*^ mutant tracheal cells than in WT cells as compared to the short axis, which is not affected (F). The median aspect ratio (long/short axis) (G) and the median apical surface area (H) are significantly smaller in *apn*^*1*^ mutant tracheal cells than in WT cells. For box plot measurements, the *n* values were calculated from 48 cells (WT) and 58 cells (*apn*^*1*^).

A striking defect observed in *apn*^*1*^ mutant larvae was the reduction in the length of the dorsal trunk (Fig 3E–3H). To determine the cellular basis of this phenotype we stained for *D*Ecad to visualize the cell outline. We could not detect significant differences in cell number within different metameres. This led us to hypothesize that shortening of tracheal tubes is caused by defective apical cell surface expansion. Therefore, we measured the long and the short axes of cells (referred to as axial and circumferential length, respectively) (see Fig 4A) as well as their cell surface area. While the circumferential cell length was not significantly different, the axial cell length of *apn*^*1*^ mutants was reduced in comparison to that of wild type cells (Fig 4A, 4B, 4E and 4F). This difference was also reflected by a reduced aspect ratio of the two axes (axial to circumferential length) (Fig 4G) and the overall reduction of the apical surface area (Fig 4C, 4D and 4H). From these results we conclude that Apn is required for anisotropic apical surface expansion and hence tracheal tube elongation.

### Apnoia is required for maintenance of Crumbs on the apical membrane of tracheal cells

**Fig 5:**
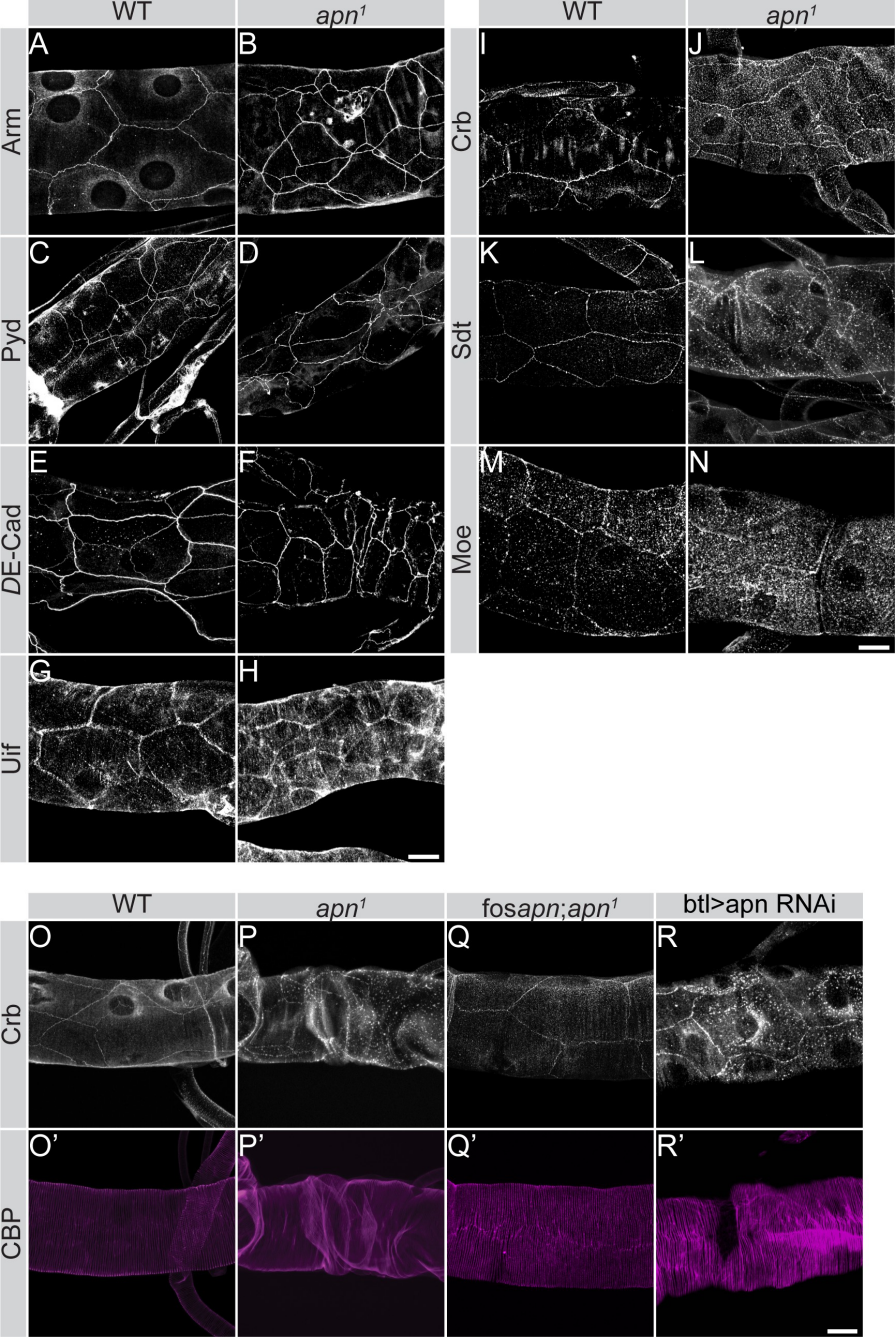
Apn-dependent tube growth correlates with defects in localization of components of the Crb complex. **(A-N)** Projections of confocal sections of tracheal dorsal trunks of second instar larvae. *apn*^*1*^ mutants stained for the adherens junction proteins Arm (B), Polychaetoid (Pyd; D), *D*E-Cad (F) and for the apical protein Uif (H) do not display any significant changes compared to the respective wild type (WT) tissues (A, C, E, G). In contrast, localization of the Crb complex proteins, Crb (I, J) and Sdt (K, L), and the FERM protein Moe (M, N) are mis-localized in *apn*^*1*^ mutants. Scale bars: 20μm. **(O-R’)** Tracheae of second instar larvae stained for Crb (O-R) reveal strong reduction of Crb from the apical membrane and its accumulation in cytoplasmic vesicles in *apn*^*1*^ mutants (P) and upon tracheal knockdown of *apn* (R). Staining for chitin binding probe (CBP; O’-R’) reveals twisted tracheal tubes in *apn*^*1*^ mutants (P’) and upon tracheal knockdown of *apn* (R’), though less severe. Expression of an additional copy of *apn* (fosapn_mCherry.NLS_) rescues Crb apical localization (Q) and tubular structure defects (Q’). Scale bar: 20μm.

Regulation of apical cell surface area during axial growth of tracheal tubes has been shown to require junctional and polarity proteins as well as the apical protein Uif [55–58]. Therefore, to better understand the mechanism by which *apn* ensures apical membrane growth, we examined the subcellular distribution of junctional and polarity proteins in the tracheae of *apn*^*1*^ mutants. The AJ markers Armadillo (Arm), the *Drosophila* β-catenin [59] (Fig 5A and 5B), Polychaetoid (Pyd), the single *Drosophila* ZO-1 orthologue [60] (Fig 5C and 5D) and *D*Ecad (Fig 5E and 5F) localized similar to the wild type tracheae. *apn*^*1*^ mutant tracheal cells also showed normal distribution of Uif (Fig 5G and 5H). These results indicate no major defects in apico-basal polarity and epithelial integrity of the tracheal tube in *apn*^*1*^ mutant larvae. The physical interaction between Apn and Crb motivated us to analyze the expression of Crb in *apn*^*1*^ mutants. In wild type tracheal cells of second instar larvae, Crb is localized in the subapical region, outlining the cell (Fig 5I). In contrast, Crb strongly accumulated in cytoplasmic vesicles of multicellular, autocellular and seamless tubes in *apn*^*1*^ mutant tracheae and upon knock-down of *apn* (Fig 5J and 5R and S4A–S4D’ Fig). Consistent with these results, not only multicellular, but also autocellular and seamless tubes were twisted and uninflated (S4E–S4F’ Fig). However, the total protein levels of Crb were unchanged as revealed by western blotting (S2F Fig). To investigate whether *apn* is required for Crb apical localization only in the trachea, we analyzed another epithelial tube, the salivary gland. A uniform apical localization of Crb was observed in both wild type and *apn*^*1*^ mutant salivary glands, indicating a tracheae-specific role of *apn* (S4H–S4I’ Fig).

Similar to Crb, Stardust (Sdt) (Fig 5K and 5L) and Moesin (Moe) (Fig 5M and 5N), whose subapical localization depend on Crb in many epithelia [61–64], are found in the same vesicular compartments as Crb. The introduction of one copy of the *apn* genomic locus (fos*apn*_mCherry.NLS_) into the *apn*^*1*^ mutant background restored Crb membrane localization and suppressed the accumulation of Crumbs loaded vesicles (CLVs) (Fig 5O–5Q). These results indicate that Apn is required for Crb trafficking to or maintenance at the plasma membrane of tracheal cells.

**Fig 6:**
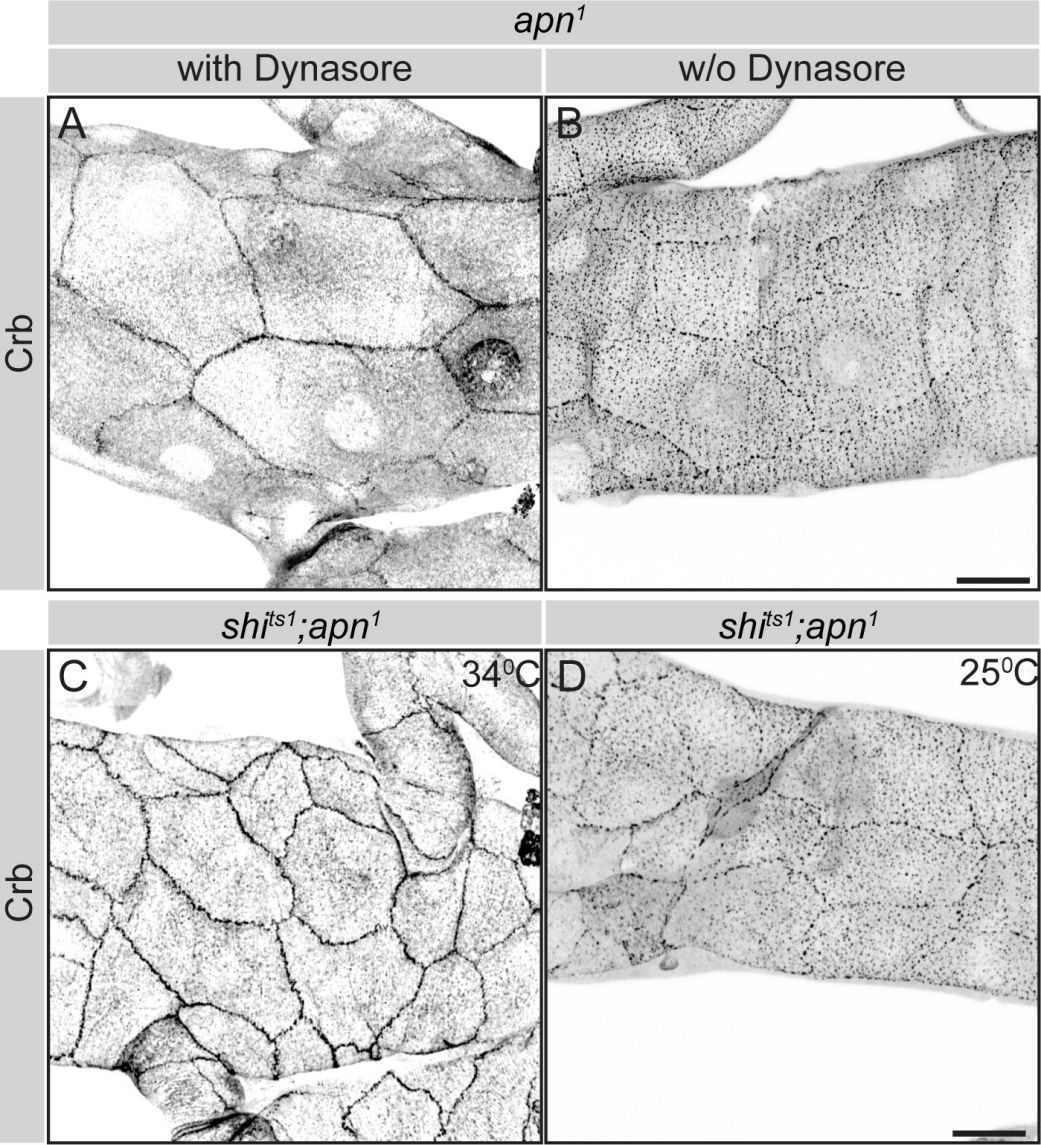
Blocking endocytosis prevents internalization of apical Crb in *apn*-mutant tracheae. (**A, B**) *apn*^*1*^ mutant tracheal tubes stained for Crb, after 2 hours incubation in 60μM dynasore (A) or in the absence of dynasore (B). (**C, D**) Tracheal tubes hemizygous for the temperature sensitive *dynamin* allele, *shibire*^*ts1*^ stained for Crb, after 4 hours incubation at 34°C (C) and 25°C (D, as control). Scale bars: 20μm.

In order to distinguish between these two possibilities, we blocked endocytosis in *apn*^*1*^ mutant tracheae chemically and genetically. After 2 hours incubation with dynasore, an inhibitor of Dynamin [65], Crb was mostly localized at the plasma membrane in *apn*^*1*^ mutant tracheal cells (Fig 6A). In contrast, *apn*^*1*^ mutant cells incubated with dynasore-free medium showed only punctate staining of Crb (Fig 6B). To corroborate this result, we blocked endocytosis by using *shibire*^*ts1*^ (*shi*^*ts1*^), a temperature-sensitive allele of *shi*, which encodes Dynamin. When incubated at the restrictive temperature (34°C) *shi*^*ts1*^*;apn*^*1*^ double mutant tracheae retained Crb at the apical plasma membrane (Fig 6C), as compared to *shi*^*ts1*^*;apn*^*1*^ mutant tracheae, incubated at the permissive temperature (25°C) (Fig 6D). From these results we concluded that Apn is required for Crb maintenance at the apical membrane.

**Fig 7:**
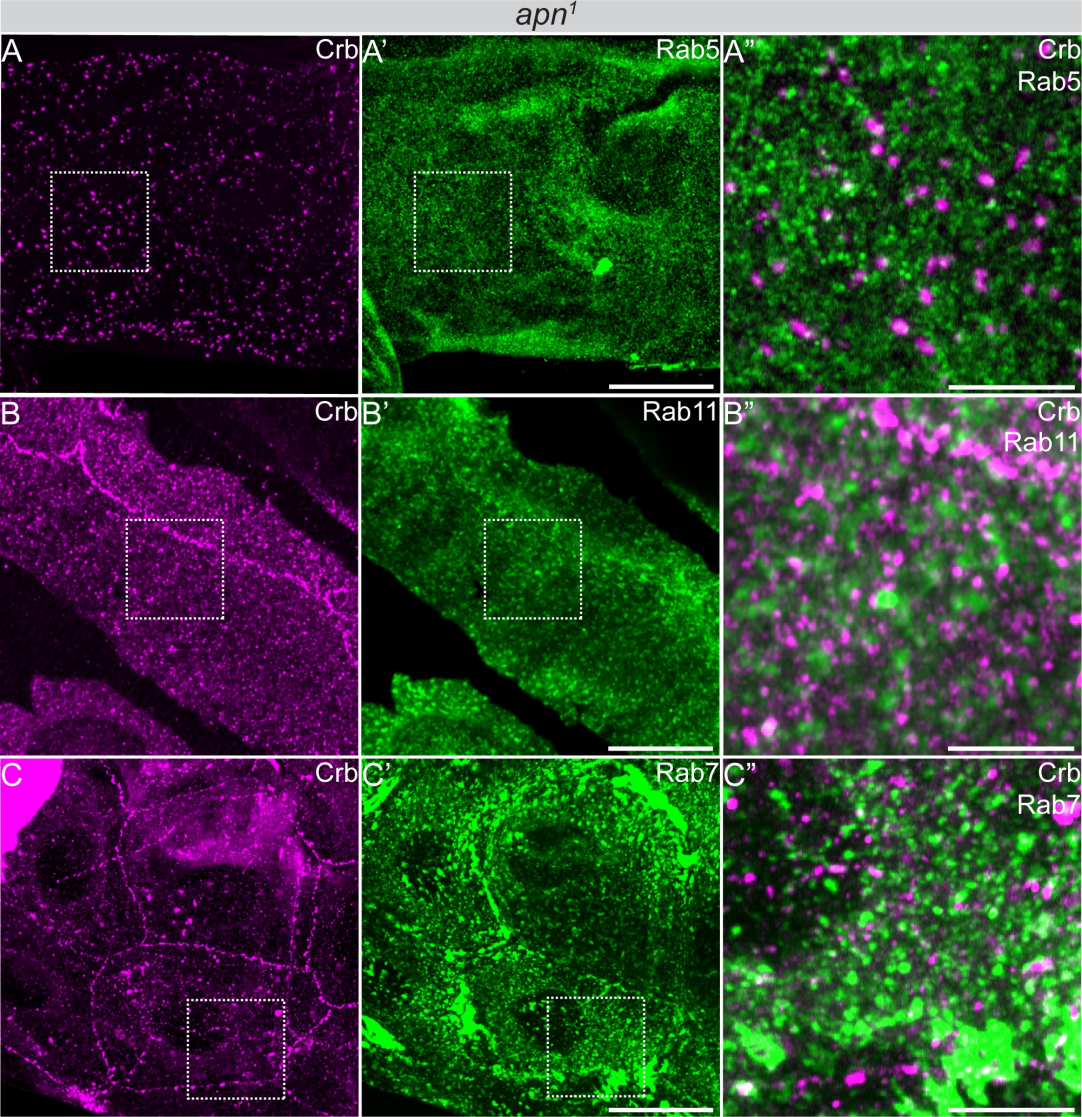
Crb subcellular accumulation in *apn*^*1*^ mutant tracheal cells. Projections of confocal sections of second instar tracheal tubes of transgenic lines expressing the respective YFP-tagged Rab protein stained for Crb (magenta) or YFP (green). (A”, B”, C”) show magnifications of the boxed area in (A-A’, B-B’, C-C’), respectively. (**A**, **A’**) *apn*^*1*^ mutant tracheal tubes immunostained for Crb and Rab5-YFP. (A”) Magnification shows vesicular Crb, most of which does not colocalize with Rab5. (**B**, **B’**) *apn*^*1*^ mutant tracheal tubes immunostained for Crb and Rab11-YFP. Magnification (B”) shows vesicular Crb, most of which does not colocalize with Rab11. (**C**, **C’**) *apn*^*1*^ mutant tracheal tubes immunostained for Crb and Rab7-YFP. Magnification (C”) shows vesicular Crb colocalization with Rab7 of approx. 25%. Scale bars: A, A’, B, B’, C, C’: 20μm and A”, B”, C”: 5μm.

**Fig 8:**
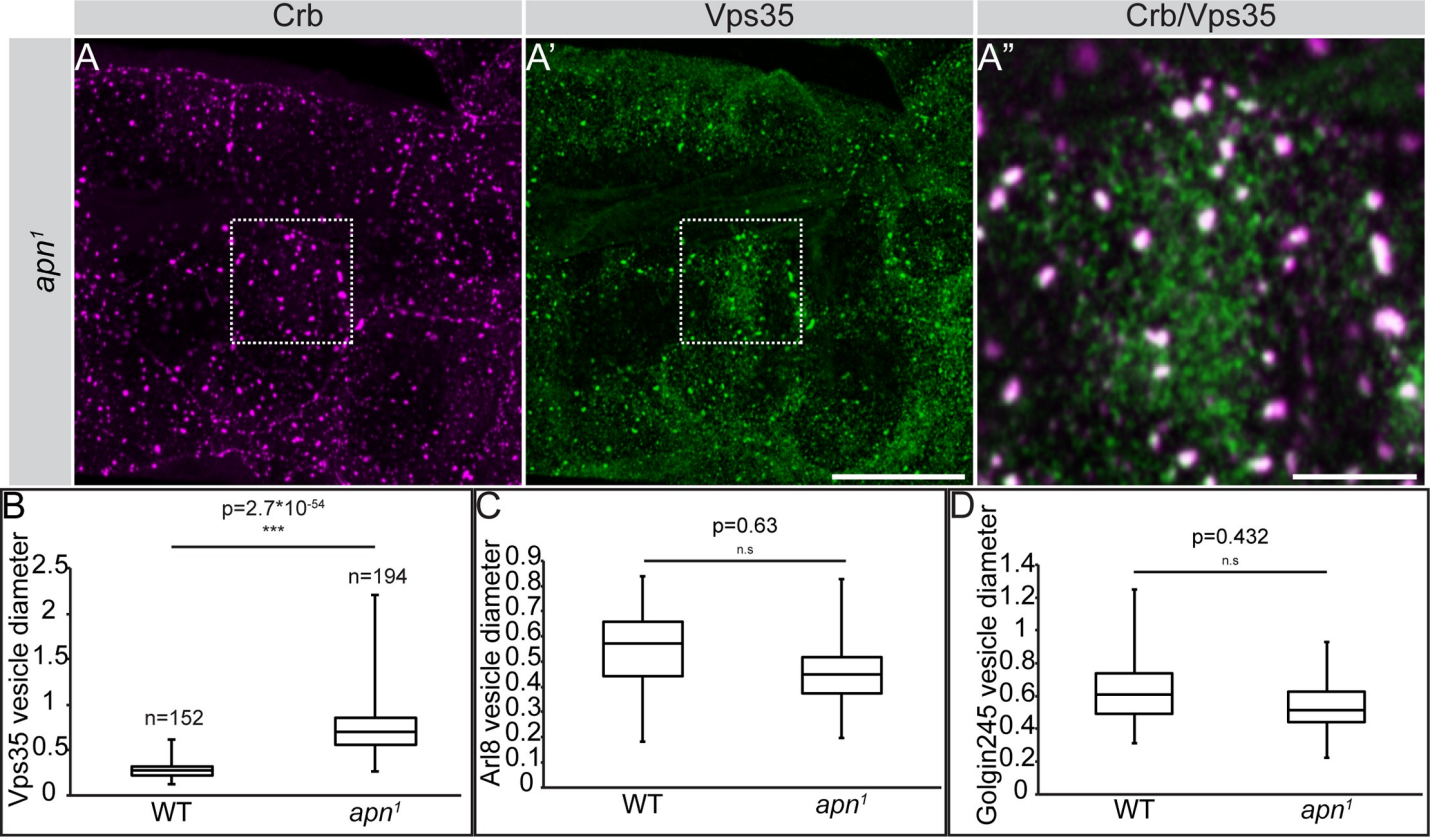
Crb is localized in abnormally large, Vps35-positive vesicles in *apn*^*1*^ mutant tracheal cells. (**A-A”**) Projections of confocal sections of second instar tracheal tubes, stained for Crb (magenta) and Vps35 (green). (A”) shows a magnification of the boxed area in A and A’. A substantial number (approx. 79%) of Crb-positive vesicles are also positive for Vps35 in *apn*^*1*^ mutant tracheal cells. Scale bars: A, A’: 20μm and A”: 5μm. (**B-D**) Quantification of the size of Vps35-positive vesicles (B), Arl8-positive vesicles (C) and Golgin245-positive vesicles in *apn*^*1*^ and wild type (WT) tracheal cells. Only Vps35-positive vesicles show a significant increase in size.

A striking cellular phenotype of the *apn*^*1*^ mutants is the accumulation of Crb in intracellular vesicles (Fig 5I and 5P). To determine their identity, we analyzed components of the trafficking machinery, including markers for endosomes, lysosomes and retromer. No major co-localization was observed between CLVs and the early endosomal markers Rab5 (Fig 7A–7A”) and Hrs (S5A–S5A” Fig) or Rab11, a marker for the recycling endosome (Fig 7B–7B”). Interestingly, 25% of CLVs were also positive for the late endosomal marker Rab7 (Fig 7C–7C”). No major overlap was found between vesicular Crb and Lamp1 [66] or Arl8 [67], two markers of the lysosome (S5B–S5B” and S5C–S5C” Fig). Strikingly, about 79% of CLVs co-localized with the retromer component Vps35 (Fig 8A–8A”).

We noticed that the majority of Vps35-positive vesicles were significantly larger in *apn*^*1*^ mutants, measuring around 0.7μm (n = 194 vesicles) in diameter, as compared to 0.27μm (n = 152 vesicles) in control larval tracheal cells (Fig 8B). No significant size differences in two other trafficking compartments, such as the Arl8- (lysosomal) and the Golgin245- (*trans-*Golgi) [68] positive vesicles, were observed between the two genotypes (Fig 8C and 8D). This result suggests that the size increase specifically in the Vps35-positive compartment is an aspect of the *apn*^*1*^ mutant phenotype.

Taken together, these results suggest that Apn maintains apical Crb by preventing its clathrin-dependent endocytosis. Loss of *apn* results in Crb accumulation in Vps35/retromer-positive vesicles of increased size.

### Tracheal defects caused by *apn* depletion are mediated by *crb*

**Fig 9:**
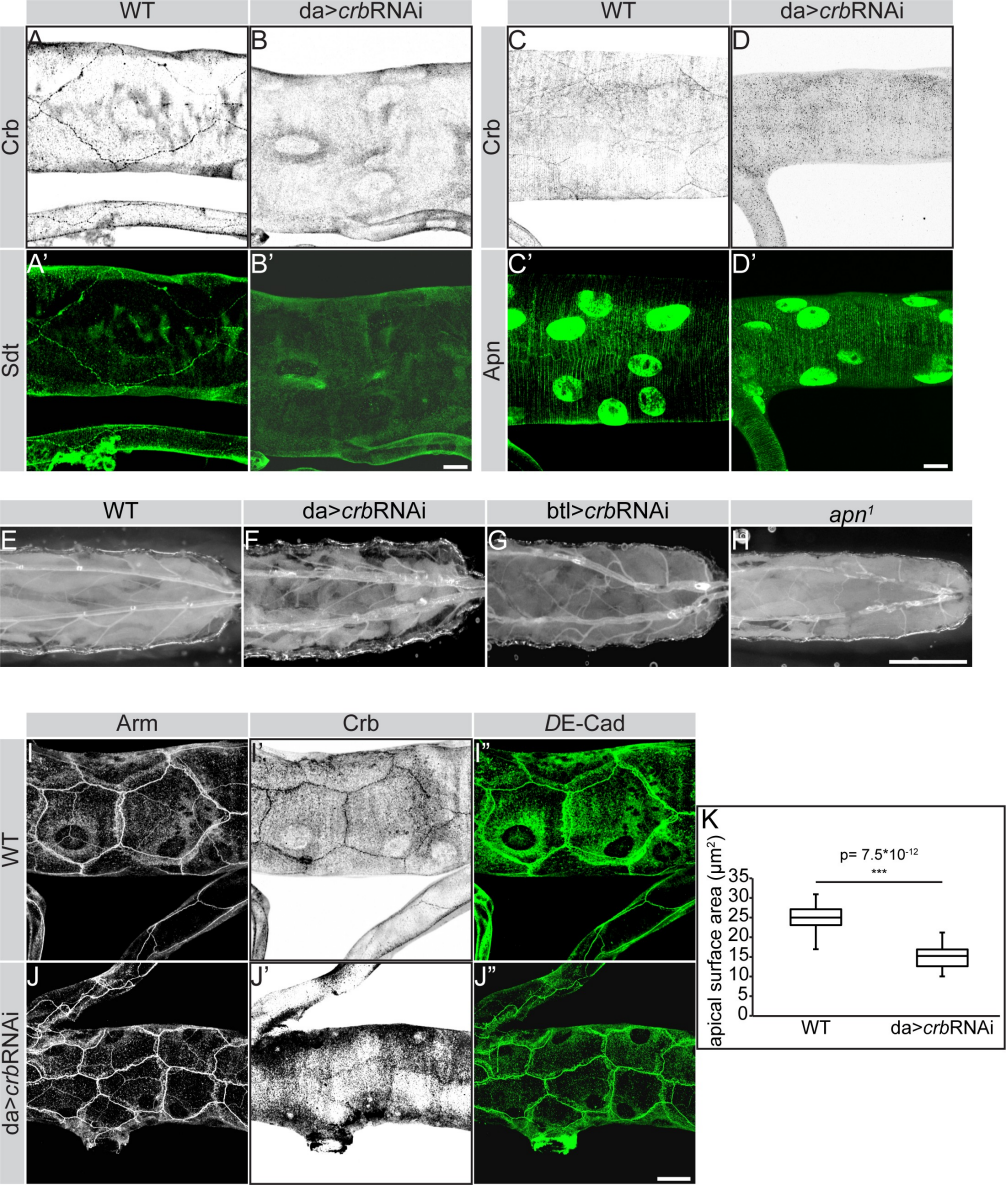
Downregulation of *crb* induces tracheal tube defects similar to those of *apn*^*1*^ mutant tubes. (**A-D’**) RNAi-mediated downregulation of Crb (B, B’ and D, D’) results in strong depletion of Crb (B, D) and Sdt (B’), but does not affect Apn expression (D’) in the dorsal trunk of second instar larvae. Projections of confocal sections of second instar tracheal tubes, stained for Crb (A-D), Sdt (green; A’, B’) and Apn (green; C’, D’). Nuclear staining in C’ and D’ is due to unspecific staining of the Apn antibody. Scale bars: 20μm. (**E-H**) Brightfield images of second instar larvae showing the tracheal tubes. Tracheal tubes are rigid and gas filled in wild type (WT) (E). Reduction of Crb using either a ubiquitous (F) or a tracheal-specific (G) Gal4 recapitulates the characteristic *apn*^*1*^ mutant tracheal defects (compare F, G and H), with constricted and gas-deficient tubes. Scale bars: 20μm. (**I-J”**) RNAi-mediated downregulation of *crb* strongly reduces Crb (compare I’ and J’) but does not affect the localization of Arm or *D*Ecad (compare I’ with J’ and I” with J”, respectively). Scale bars: 20μm. **(K)** Quantification of the median apical surface area of *crb* RNAi tracheal cells. The surface area is significantly reduced compared to that of WT tracheae.

Since loss of apical Crb is often associated with reduced apical membrane [28,69,70] and Crb is depleted from the apical membrane in *apn*^*1*^ mutant tracheal cells, we asked whether the impaired apical surface growth observed in *apn*^*1*^ mutant tracheae is due to its effect on apical Crb. Since homozygous *crb* mutant embryos die with severe defects in many epithelia, including the tracheae [24,71], we knocked-down *crb* in tracheal tubes by expressing *crb* RNAi ubiquitously (using *da*-Gal4) or specifically in the tracheae (using *btl*-Gal4). This resulted in a strong depletion of Crb and its binding partner Sdt (Fig 9A, 9B, 9A’ and 9B’), but had no effect on Apn expression and localization (Fig 9C, 9D, 9C’ and 9’D). RNAi-mediated downregulation of *crb* reproduced several aspects of the *apn*^*1*^ mutant phenotypes, such as twisted tracheal tubes, lack of gas filling (Fig 9E–9H) and reduced apical surfaces of tracheal tube cells (Fig 9I–9K). No defect in apico-basal polarity was observed upon knockdown of Crb (Fig 9I, 9J, 9I” and 9J”). In addition, most animals died at L2 (larval stage 2) with some surviving until L3 (larval stage 3).

**Fig 10:**
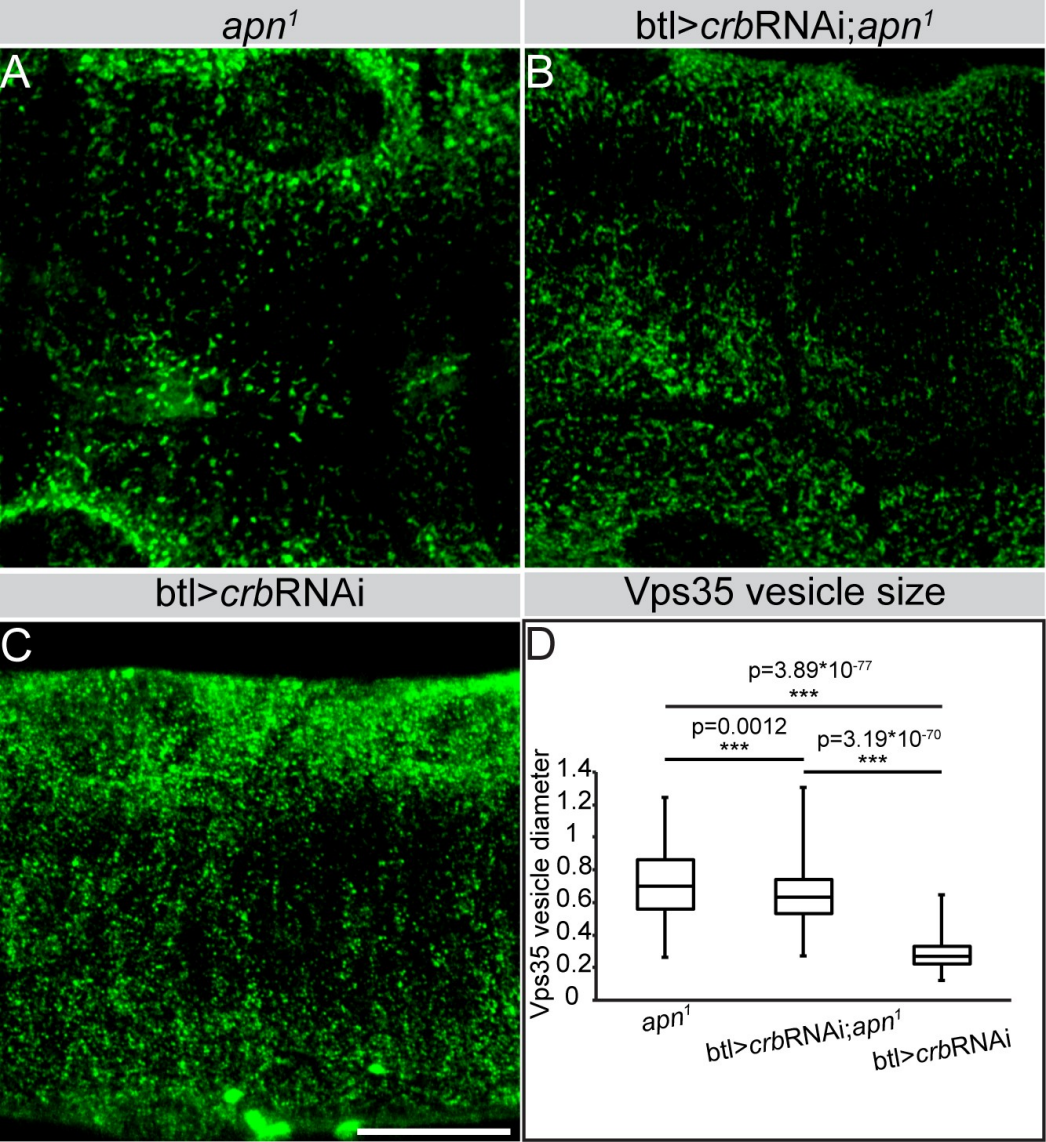
RNAi-mediated knockdown of *crb* reduces the size of Vps35-positive vesicles in *apn*^*1*^ mutant tracheal cells. (**A-C**) Projections of confocal sections of second instar tracheal tubes, stained for Vps35 (green). Scale bars: A-C: 20μm. (**D**) Quantification of the size of Vps35-positive vesicles in tracheal *crb* knockdown, *apn*^*1*^ mutants and *apn^1^* mutant upon *crb* knockdonw.

To assess whether the increased size of Vps35 positive vesicles in *apn*^*1*^ mutants are due to Crb accumulation in these vesicles, we knocked-down *crb* in *apn*^*1*^ tracheae using *btl*-Gal4. We found a small, yet significant, reduction in the size of Vps35 positive vesicles in *apn*^*1*^ tracheal cells upon *crb* RNAi expression, compared to that of *apn*^*1*^ single mutants (Fig 10A, 10B and 10D and S6A–S6C Fig). In contrast, Vps35 positive vesicles in tracheal cells expressing *crb* RNAi in otherwise wild-type animals are comparable in size to those of wild type Vps35 vesicles (compare Fig 10C and 10D and Fig 8B).

These results are the first to show that loss of *crb* results in a reduction of the apical surface area of larval tracheal cells, which in turn prevents proper tube elongation. In addition, they identify Apn as a novel regulator of apical Crb in the developing tracheae, which controls dorsal trunk maturation and expansion. Absence of *apn* leads to accumulation of Crb in Vps35 positive vesicles, which may contribute to the increase in vesicular size.

## Discussion

This work identifies Apn as an essential protein for airway maturation in *Drosophila* larval stages. Apn is localized apically in tracheal epithelial cells, where it co-localizes and physically interacts with Crb. *apn*^*1*^ mutant larvae exhibit loss of tracheal tissue structure, manifested by tube size defects and impaired gas filling, resulting in body size reduction and lethality at second instar. At the cellular level, exclusion of Crb from the apical membrane in *apn*^*1*^ mutant larval tracheae goes along with apical cell surface reduction and an overall tracheal tube shortening. Absence of *apn* leads to Crb inhibition and accumulation in enlarged, Vps35/retromer-positive vesicles.

Elongation of the tracheal tube has been extensively studied in embryos where it has been shown to rely on different mechanisms, such as the organization of the aECM and cell shape changes [33,72–76]. Anisotropic growth of the apical plasma membrane is an additional mechanism to achieve proper longitudinal tube expansion. However, only few proteins have been described so far to regulate this process. One of these, the protein kinase Src42A, is required for the expansion of the cells in the axial direction, and loss of *Src42A* function results in tube length shortening, which is associated with an increased tube diameter [72,75,77]. Src42A has been suggested to exert its function, at least in part, by controlling *D*Ecad recycling and hence adherens junctions remodeling [72] and/or by its interaction with the Diaphanous-related formin *d*DAAM (*Drosophila* Dishevelled-Associated Activator of Morphogenesis), loss of which results in reduced apical levels of activated pSrc42A [75]. More recently, Src42A has been suggested to control axial expansion by inducing anisotropic localization of Crb preferentially along the longitudinal junctions [78]. However, we never observed any anisotropic distribution of Crb in wild type larval tracheal cells, making it unlikely that, at this developmental stage, axial expansion is regulated by a Src42A-dependent mechanism. This assumption is corroborated by the observation that, unlike in *Src42A* mutants, the lack of longitudinal expansion in *apn*^*1*^ mutant larval tubes is not associated with circumferential expansion. Another protein regulating tube elongation in the embryo is the epidermal growth factor receptor, EGFR. Expressing a constitutively active EGFR results in shortened tracheal tubes with smaller apical cell surfaces, but with increased diametrical growth. In this condition, Crb shows altered apical distribution [78,79]. This phenotype differs from the *apn*^*1*^ phenotype, where apical localization of Crb is almost completely lost and only longitudinal tube growth is affected. This suggests that Apn executes tube length expansion by a different mechanism.

How does decrease in tubular growth lead to loss of tracheal structure? During development, the larval body, including the tracheal tissue, elongates about 8-fold [18]. Impaired axial tracheal cell growth in *apn*^*1*^ mutants thus may affect the balance between the forces exerted by apical membrane growth on the one hand and the resistance provided by the luminal aECM on the other, an important mechanism described previously to control tube shape in the embryo [33]. This could lead to physical rupture of tubes mutant for *apn*^*1*^, allowing fluid entry. The presence of fluid would, in turn, disrupt proper gas filling, resulting in hypoxia and, consequently, in impaired body growth.

**Fig 11:**
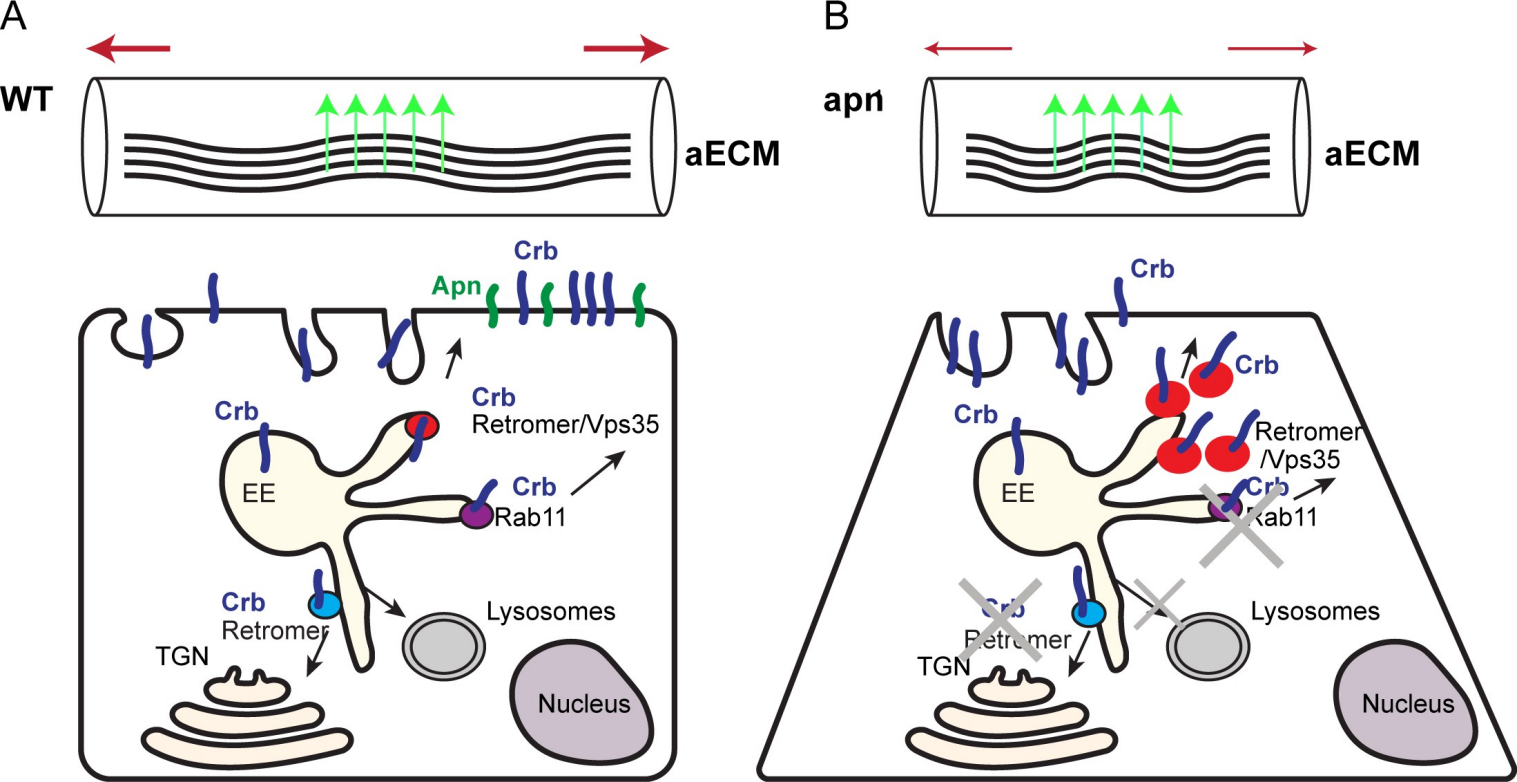
Model to explain Apn-mediated larval tracheal tube growth. (**A**) Top: In the wild type (WT) dorsal trunk, the apical membrane grows along the axial axis (thick red arrows) and pulls the apical extracellular matrix (aECM), until the aECM resistance (indicated by green arrows) balances the forces provoked by tube elongation [33]. Bottom: In WT tracheal cells, Crb (blue) is enriched at the apical membrane where it controls apical surface growth. Apn (green) in the apical membrane is responsible for Crb maintenance and therefore ensures tube elongation. Crb trafficking involves recycling by the retromer to either the trans-Golgi network (TGN; blue vesicles) or to the plasma membrane (red vesicles), or by Rab11, or its degradation in the lysosome. (**B**) Top: In the *apn*^*1*^ mutant dorsal trunk, the pulling forces of the apical membrane expansion are decreased due to decreased growth of the apical membrane (thin red arrows), whereas the forces mediated by the aECM are likely to remain unchanged, causing breakage of the tube. Bottom: In the absence of Apn, Crb is depleted from the apical surface due to increased endocytosis. Crb is trapped in enlarged, Vps35 (retromer)-positive vesicles. It fails to be recycled (as shown by the lack of colocalization with Rab11), but is also not degraded, pointing to a functional defect of the retromer.

Several studies have shown that, in some tissues, Crb accumulation on the apical membrane is mediated by the retromer complex, which controls either the retrograde transport of Crb to the *trans*-Golgi [80] or the direct trafficking from the endosomes to the plasma membrane [39,41,79]. The physical interaction of Apn and Crb, the functional requirement of Apn for Crb apical localization and the fact that in *apn*^*1*^ mutants Crb is trapped in Vps35-positive/retromer vesicles all suggest that Apn is required for trafficking and/or maintenance of Crb at the apical membrane (Fig 11).

However, the increase in the size of Vps35-positive vesicles in *apn*^*1*^ mutant cells, which is, to some extent, due to the accumulation of Crb, suggests defects in retromer function, which may prevent Crb lysosomal degradation. Further studies will help to elucidate at which level Apn controls Crb trafficking in larval tracheae.

## Materials and methods

### Fly stocks

Flies were maintained at 25°C with 50% humidity unless stated otherwise. The fos*apn*_sfGFP_ (tagging with 2XTY1-SGFP-V5-preTEV-BLRP-3XFLAGdFRT was done C-terminally) and fos*apn*_mCherry.NLS_ (tagging with ubi-mCherry-NLS-T2A was done N-terminally) were provided by the Flyfos library at MPI-CBG [45]. The following fly lines were used: Rab5-YFP, Rab7-YFP and Rab11-YFP (http://rablibrary.mpi-cbg.de/cgi-bin/rab_overview.pl) [81] (kindly provided by Marko Brankatschk), UAS-moe-GFP (kindly provided by Brian Stramer) [82], UAS-ANF-mCherry (modified from [52]), and w; *D*Ecad::GFP [83], w; Sas:: Venus [47], w; btl-Gal4 [84]. The following fly stocks were obtained from the Bloomington *Drosophila* Stock Center (BDSC): y[1] M{w[+mC] = nos-Cas9.P}ZH-2A w[*] (BDSC, #54591), *shi*^*ts1*^ (BDSC, #7068), y[1] v[1]; P{y[+t7.7] v[+t1.8] = TRiP.HMS01842}attP40 (*crb*^*RNAi*^) (BDSC, #38373), w[1118];da^G32^-Gal4 (BDSC, #55851) [21], w[1118]; Df(3R)Exel8158/TM6B, Tb (BDSC, #7974) and UAS-lamp1-GFP (BDSC, #42714). The CG15887 (*apn*^*RNAi*^) (VDRC, #9070) was obtained from the Vienna *Drosophila* Resource Center.

### Generation of *apn*^*1*^ mutant allele by CRISPR/Cas9

Target sites were designed using the settings of the flyCRISPR Optimal Target Finder (http://tools.flycrispr.molbio.wisc.edu/targetFinder/), to guide Cas9 to two target sites, one at the 5’UTR (gagggtctgggccggcttacTGG) and one at the 3’UTR end (gcaaagtcacggagaaatctGGG) (UPPERCASE: PAM) of CG15887 (*apn*), with the aim to delete the whole Open Reading Frame. The following phosphorylated DNA oligomers were used as primers for PCR, using 10ng of pCFD4-U6:1U6:3tandem gRNAs as template (Addgene #49411;[85]): forward primers 5’-[P]-tatataggaaagatatccgggtgaacttcgGCAAAGTCACGGAGAAATCTgttttagagctagaaatagcaag-3’, reverse primer 5’-[P]-attttaacttgctatttctagctctaaaacGTAAGCCGGCCCAGACCCTCcgacgttaaattgaaaataggtc-3’. The resulting DNA fragment was cloned into pCFD4-U6:1U6:3tandemgRNAs via Gibson Assembly after linearization of the vector with BbsI. To replace the CG15887 ORF with 3XP3-dsRed we used the pHD-DsRed-attP vector (Addgene #51019;[86]). The homology arms necessary to obtain Homology Directed Repair were sequences covering 1kb regions of upstream and downstream of 5’UTR and 3’UTR gRNA cut sites, respectively. Cloning into the vector was obtained with AarI for the 5’-homology arm (5’-HA) and SapI for the 3’-homoly arm (3’-HA). Primer sequences are: Forward primer 5’-HA (5’-TGTACACCTGCGAATTCGCCCACACTGTTTGGCATCTGGCGGCGCTCCTCC-3’), Reverse primer 5’-HA (5’-TGTACACCTGCAGATCTACTTTCTCCGTGACTTTGCTCATAGCTCATTATGG-3’), Forward primer 3’-HA (5’-GCTAGCTCTTCGTATTACTGGGCGGCTACTTGAAATTCGGGAGCC-3’), Reverse primer 3’-HA (5’- GCTAGCTCTTCGGACCCCCAATAACATGTCCGTCCGCACTACG-3’). Homology arm sequences were amplified from the BAC genomic clone BACR05K08 (obtained from BACPAC resources center (BPRC). The two plasmids were injected in a concentration of 400ng each, into nos::Cas9 embryos [85].

### Generation of transgenic flies

To generate the UAS-*apn* transgenic line, the CG15887 (*apn*) cDNA (RE53127; obtained from DGRC) was cloned into pJFRC-MUH-mCitrine[87] by BglII-NotI using standard molecular biology techniques. Plasmid constructs were injected by BestGene.

### Dynasore treatment of larval tracheae

*apn*^*1*^ mutant tracheae from second instar larvae were dissected in Grace’s medium supplemented with Pen/Strep. Tissues were incubated in 60μM dynasore (Enzo Life Sciences) in Grace’s medium containing Pen/Strep and 2.5% FCS at room temperature for 2hr. The dynasore was washed out and tracheae were fixed in 4% FA in Grace’s medium for 30min.

### Yeast-two-Hybrid screen

Part of the coding sequence of a *Drosophila melanogaster crb* cDNA (encoding aa: 2034–2189) (GenBank accession number NM_001043286.1) was PCR-amplified and cloned in pB102, in frame with the STE2 leader sequence at the N-terminus and ubiquitin (Cub) at the C-terminus of the bait which is coupled to the artificial transcription factor LexA-VP16 (STE2-Crb-Cub-LexA-VP16). The construct was verified by sequencing. Prey fragments were isolated from a MBmate screen with *Drosophila melanogaster* Crb as bait against a *Drosophila* Embryo NubG-x (D3DE_dT) library (NubG stands for the N-terminal domain of mutated ubiquitin and x for the prey fragment). Interaction pairs were tested in duplicate as two independent clones. For each interaction, several dilutions (undiluted, 10^−1^, 10^−2^, 10^−3^) of the diploid yeast cells (culture normalized at 5x10^7^ cells) and expressing both bait and prey constructs were spotted in selective media. The DO-2 selective medium lacking tryptophan and leucine was used to control for growth and to verify the presence of both the bait and prey plasmids. The different dilutions were also spotted on a selective medium without tryptophan, leucine and histidine (DO-3). Six different concentrations of 3-AT, an inhibitor of the HIS3 gene product, were added to the DO-3 plates to increase stringency and reduce possible auto-activation. The following 3-AT concentrations were tested: 1, 5, 10, 50, 100 and 200mM. The 1-by-1 Yeast two-hybrid assays were performed by Hybrigenics Services, S.A.S., Paris, France (http://www.hybrigenics.com).

### Cell culture and transfection

*Drosophila* S2R^+^ cells were cultured at 25°C in Schneider's *Drosophila* medium (Sigma) supplemented with 10% fetal bovine serum. p*Act5*-Gal4 together with UAS-apn^FL^ and/or UAS-crb^FL^ [21] (encoding full-length Apn and Crb, respectively) was transfected into S2R^+^ cells using FuGENE HD (Promega) according to the manufacturer's protocol.

### Immunoprecipitation

Transfected cells were harvested after 48h, washed with ice-cold PBS (120mM NaCl in phosphate buffer at pH 6.7), resuspended in lysis buffer (containing 10% glycerol; 1% Triton X-100; 1.5mM MgCl_2_; 120mM NaCl; 100mM PIPES, pH 6.8; 3mM CaCl_2_; 1 mM PMSF and Complete). Cells were lysed on ice for 20min and lysates were spinned at 14.000rpm for 20min at 4°C. The supernatant was incubated with the antibody for 2. In the meantime 50μl of Protein G were washed 3 times with blocking solution and incubated with the antibody solution overnight at 4°C. Protein G beads were collected by centrifugation for 2min at 3.000rpm and washed 4 times with lysis buffer. Beads were resuspended in 1.5x SDS sample buffer and heated for 5min at 95°C.

### Western blot

Wild-type and *apn*^*1*^ mutant embryos and dissected larval tracheae were homogenized on ice using a Dounce tissue grinder in 1mL of lysis buffer containing 130mM NaCl, 50mM Tris-HCl pH = 8, 0,5% Triton-X and protease inhibitor (Roche). After 30min at 4°C under rotation the homogenate was centrifuged for 20min at 14.000rpm. Sample buffer 3x SDS was added to the supernatant and boiled for 5min at 95°C.

Proteins were separated by SDS-PAGE and blotted onto nitrocellulose 0.45 membrane (Amersham). After blocking in 5% BSA+TBST, the membrane was incubated overnight with rabbit anti-Apn diluted 1:1000, rat anti-Crb[88] diluted 1:1000 and mouse anti-alpha-tubulin (Sigma) diluted 1:5000 in blocking buffer. Peroxidase antibodies were used for detection.

### Proximity ligation assay (PLA)

Tracheae from *fosapn*_*sfGFP*_ third instar larvae were dissected and fixed in ice cold 4% FA in PBS. Primary antibodies against GFP (rabbit anti-GFP 1:250; Invitrogen A11122) and Crb (rat anti-Crb 1:500 [88]) were added and incubated overnight at 4°C. The Duolink PLA Kit (Sigma) was used to incubate the tissue with the PLA probes PLUS and MINUS at 37°C for 1h. Ligation of the PLA oligonucleotides and amplification were performed at 37°C for 30min and 100min, respectively. Samples were mounted in Duolink mounting media and imaged using Zeiss LSM880.

### Generation of Apn antiserum

Polyclonal antibodies against CG15887 were raised in rabbits using the KLH-conjugated synthetic peptide QQAANSSDSDSDVAESC (from the N-terminal extracellular part) for immunization. Antibodies were subsequently affinity-purified using the same peptide immobilized on SulfoLink Coupling Gel (ThermoFisher #20401) and following recommendations by the manufacturer. The work was performed by the MPI-CBG Antibody Facility.

### Immunohistochemistry

Immunostainings on embryos were done as follows: embryos were dechorionated in 50% bleach for 2min and fixed for 20min in formaldehyde/heptane mixture. After devitellinization in methanol, embryos were permeabilized in 0.1% Triton X-100/PBS except for rabbit anti-Apn staining, for which embryos were permeabilized in 0.2% Saponin/PBS. After washing, embryos were incubated for 1h at RT in blocking solution [(0.5%w/v BSA in PBST/S (0.1%v/v Triton X-100) or (0.2%w/v Saponin)]. Second instar larvae were opened in PBS and fixed in 4% formaldehyde for 20min. After washing in either 0.1% Triton X-100/PBS or 0.2% Saponin/ PBS (for anti-Apn antibody staining), tracheae were dissected and incubated in blocking solution for 1h at RT. Embryos and tracheae were incubated with primary antibodies overnight at 4°C, washed and incubated with secondary antibodies for 2h at RT. Samples were mounted in Vectashield (Vector Laboratories) and imaged with LSM880 Laser Scanning Confocal Microscope (Carl Zeiss). Unless otherwise indicated, images shown are z-stack projections of sections. Images were processed with Fiji software [89]. Cell area measurements were obtained using the Fiji Freehand selection tool.

The following primary antibodies were used: rabbit anti-Apn (1:500–1:1000) (this study), rabbit or rat anti-Crb (1:1000) [21,24,88], rabbit anti-Sdt (1:1000) [61], guinea pig anti-Cont (1:1500; gift from Manzoor Bhat), guinea pig anti-Uif (1:20; gift from Robert Ward), mouse anti-Pyd (1:1000; gift from Alan Fanning), rabbit anti-Pio (1:50; gift from Markus Affolter), guinea pig anti-HRS and anti-Vps26 (1:20 and 1:1000, respectively; gift from Hugo Bellen), rabbit anti-SAS (1:500 gift from Douglas Cavener), goat anti Golgin245 (1:200, DSHB), mouse anti-Arm (1:50, DSHB, N27A1), rabbit anti-Arl8 (1:100, DSHB), rat anti-*D*Ecad (1:50, DSHB, DCAD2), mouse anti-Dlg (1:500, DSHB, 4F3), rabbit anti-GFP A11122 (1:250, Thermo Fischer), mouse anti-GFP (1:250, Roche), Chitin binding probe-633 (1:20; gift from Maria Leptin) [90]. The secondary antibodies Alexa Fluor 488, 568 and 633 (Molecular Probes) were used at 1:400 dilution.

### RNA *in situ* hybridization

DIG-labelled RNA probes were synthesized from PCR templates amplified from for a full-length *apn* (RE53127) cDNA clone. Sequence specific primers for pFLC-I vector (BDGP resources) were: M13 (-21) 5’-TGTAAAACGACGGCCAGT-3’ and M13 (REV) 5’-GGAAACAGCTATGACCATG-3’. PCR products were purified by PCR purification columns (Promega, PCR CleanUp system). *In vitro* transcription reactions were performed by mixing the PCR product with the polymerase mix, which includes T3 RNA polymerase. RNA was labelled with digoxigenin-UTP (Roche Applied Science, #11277073910). Eggs were collected on apple juice plates for 12h. Embryos were dechorionated in 50% bleach for 2min and fixed for 20min in formaldehyde/heptane mixture. After devitellinization in methanol embryos were processed for hybridization, as modified from [91].

### Electron microscopy analysis

Larvae were fixed in 2% glutaraldehyde in 0.1M PB buffer pH 7.2 for 20min at room temperature. Larvae were transferred in microcentrifuge tubes and fixed in 1% OsO_4_/2% Glutaraldehyde and then 2% OsO_4_. Further procedures were done according to the protocol described [92]. Ultra-thin sections of 0.1μm were prepared and analyzed with Tecnai 12 BioTWIN (FEI Company).

### Image analysis

We developed a Fiji script to quantify the co-localization of proteins of the trafficking machinery (e.g. retromer, lysosome, Golgi) with Crb-positive vesicles. Two channel images showing fluorescent Crb signal and protein X signal were imported into a script for the freely available Fiji software [89] and characterized as to their overlap. The plugin was tested on Fiji current version: (Fiji is just ImageJ) ImageJ 2.0.0-rc-65/1.51w. The code of the scripts and its documentation are available on the project repository (https://git.mpi-cbg.de/bioimage-informatics/Skouloudaki_et_al_Crumbs_overlap_analysis).

## Supporting information

S1 Fig Amino acid sequence alignment of the protein encoded by CG15887 (*apn*). Prank software of the homologous sequences within the insect order was used. Colored bars indicate the protein domains. SP: Signal Peptide, N’-ECD: N-terminal Extracellular Domain, TM: Transmembrane domain, ICD: Intracellular Domain, C’-ECD: C-terminal Extracellular Domain.
(TIF)

S2 Fig Apn expression in the embryonic and larval tracheae. (**A-D**) Immunostaining of embryonic (A-B’) and larval (C, D) tracheae with anti-Apn antibody shows specific tracheal staining in wild type (WT) stage 17 embryos (A) and second instar (L2) larva (C). No Apn protein can be detected in *apn*^*1*^ mutant embryos (B) and second instar (L2) larva (D). Embryos/larvae in A’, B’ were additionally stained for chitin binding probe (CBP) to highlight the luminal matrix. Scale bars: 20μm. (**E**) Yeast-two-hybrid analysis detecting interaction between the bait (Crb) and the prey (Apn). The top plate shows growth on media lacking tryptophan and leucine, used to verify co-transformation of plasmids as well as a growth control. The bottom plate shows the same dilutions spotted on medium lacking additionally histidine and is used to confirm the interaction between bait and prey. Column 1 is the positive control whereas columns 2, 3, 4 represent the negative controls (2: pB102/pP55 empty vectors, 3: pB102 empty vector/CG15887, 4: Crb/pP55 empty vector), column 5 represents the interaction between Crb and Apn. For each interaction several dilutions (undiluted, 10^−1^, 10^−2^, 10^−3^) were spotted. Interactions were tested with two independent clones (A and B). (**F**) Western blot from lysates of larval tracheae showing the expression levels of Crb and Apn in WT- and *apn*^*1*^ mutants. Tubulin is used as loading control. The 15kDa, Apn-positive band is absent in the *apn*^*1*^ mutant extract. The higher molecular weight bands are unspecific. **(G, G’)** Proximity ligation assay (PLA) between WT and *apn*^*1*^ mutant larval tracheae using Apn and Crb antibodies shows that the interaction is abolished in mutants lacking *apn* as compared to wild type. Scale bar: 20μm. (**H, I**) *apn*^*1*^ mutant embryo (H) and larva (I) derived from germline clones (M/Z; maternal/zygotic). (H) No defects were observed in the tracheal tubes of mutant embryos. Scale bar: 100μm. (I) Defects appear at second larval instar with irregular and twisted tracheal tubes (I). Scale bar: 500μm. (**J**) Brightfield image of a hemizygous second instar larva transheterozygous for *apn*^*1*^ and a deficiency that removes *apn*. Scale bar: 500μm. (**K**) Body size reduction in *apn*^*1*^ mutants and tracheal knockdown larvae as compared to WT larvae (bottom).
(TIF)

S3 Fig *apn* controls tube elongation independent of the aECM and septate junction pathway. **(A-C)** Brightfield dorsal views of second instar larvae, showing the structure of tracheal tubes of wild type (WT) (A) and tracheal-specific *apn* down-regulation (btl>*apn* RNAi), which recapitulates the *apn*^*1*^ mutant tracheal defects (B). The tube morphology defects are partially rescued by tracheal expression of Apn (*apn*^*1*^; btl>Apn). Scale bar: 500μm. (**D-F**) The 9^th^ metamer of the dorsal trunk (yellow dotted line) of *apn*^*1*^ mutant second instar larvae (E) is shorter than that of WT larvae (D). Tracheal expression of a transgene (*apn*^1^;*btl>apn*) rescues the metamer elongation defects of *apn* mutant larvae (F). Anterior is to the left. Scale bar: 200μm. (G-H’) Transmission electron micrographs of cross sections through a WT (G, G’) and *apn*^*1*^ mutant (H, H’) second instar trachea. (**G–H**) Axial views of the dorsal trunk (DT), G’ and H’ are higher magnifications to depict the larval cuticular ECM (epi- and procuticle) and the taenidial ridges. Scale bars: G, H 7.5μm; G’, H’ 700nm. (**I-J’**) Immunostaining of larval tracheal tubes with antibodies against the apical extracellular matrix (aECM) proteins Dumpy (Dp) (I, J) and Piopio (I’, J’). Scale bars: 20μm. (**K-L’**) Tracheal maturation of WT (K, K’) and *apn*^*1*^ mutant (L, L’) second instar larvae. Secretion of the luminal protein ANF-Cherry (E, F), as well as its clearance from the luminal space (K’, L’), are comparable between WT and *apn*^*1*^ mutants. Scale bars: 50μm. (**M-N’**) Immunostaining of WT and *apn*^*1*^ mutant tracheal tubes of second instar larvae with antibodies against the septate junction proteins Contactin (Cont) (M, N) and Discs Large (Dlg) (M’, N’). Scale bar: 20μm.
(TIF)

S4 Fig Distribution of Crb in tracheal branches of distinct cellular architecture and in salivary glands. **(A-B‴)** Confocal projections showing tracheal tubes of wild type (WT, A-A’”) and *apn*^*1*^ mutant (B-B‴) second larval instar larvae, stained with anti-Crb. Crb localization is affected in multicellular tubes (MT), lateral branches [autocellular (AT) and seamless tubes (ST)] of mutant larvae. Scale bars: (A, B, A”, B”) 20μm and (A’, B’, A”‘, B”‘) 10μm. (**C-D’**) RNAi-mediated knockdown of *apn* by *daughterless*-Gal4 (*da*-Gal4) results in accumulation of Crb-positive cytoplasmic punctae (compare C and D) and strong reduction of Apn (compare C’ and D’). Nuclear Apn signal is considered to be unspecific. Scale bars: 20μm. (**E-F’**) Brightfield lateral views of second instar larvae showing the structure of multicellular tubes (MT), autocellular (AT) and seamless tubes (ST) of WT (E, E’) and *apn*^*1*^ mutants (F, F’). Scale bars: E, F 200μm; E’, F’ 1000μm. (**G-H’**) Confocal projections showing the salivary gland of WT (G, G’) and *apn*^*1*^ mutants (H, H’) second instar larvae, stained for Crb and Dlg. Scale bars: 20μm.
(TIF)

S5 Fig Endosomal sorting components in *apn^1^* mutants. (**A-A”**) *apn*^*1*^ mutant tracheal tubes of second instar larvae immunostained for Crb (magenta) and Hrs (green). Magnification in A” shows hardly any co-localization of vesicular Crb and Hrs. (**B-B”**) *apn*^*1*^ mutant tracheal tubes of second instar larvae immunostained for Crb (magenta) and Lamp1 (green). Magnification in B” shows hardly any co-localization of vesicular Crb and Lamp1. (**C-C”**) *apn*^*1*^ mutant tracheal tubes of second instar larvae immunostained for Crb (magenta) and Arl8 (green). Magnification in C” shows hardly any co-localization of vesicular Crb and Arl8 staining. Scale bars: A, A’, B, B’, C, C’ 20μm; A”, B”, C” 5μm.
(TIF)

S6 Fig Expression of Crb of *apn^1^* and *crb* depletion tracheal cells. **(A-C’)** RNAi-mediated downregulation of Crb (A, C) results in depletion of Crb, but does not affect Dlg expression (A’, C’) in the dorsal trunk of second instar larvae. In *apn*^*1*^ mutants, Crb is detected in cytoplasmic punctae (B), whereas Dlg is properly localized baso-laterally in tracheal cells (B’). Projections of confocal sections of second instar tracheal tubes, stained for Crb (A-C), Dlg (green; A’-C’). Scale bars: 20μm.
(TIF)
