# Supplementary figures and images for: The apical protein Apnoia interacts with Crumbs to regulate tracheal growth and inflation
Source: PLoS Genet. 2019 Jan 15;15(1):e1007852. doi: 10.1371/journal.pgen.1007852 (PMC6333334; doi:10.1371/journal.pgen.1007852)

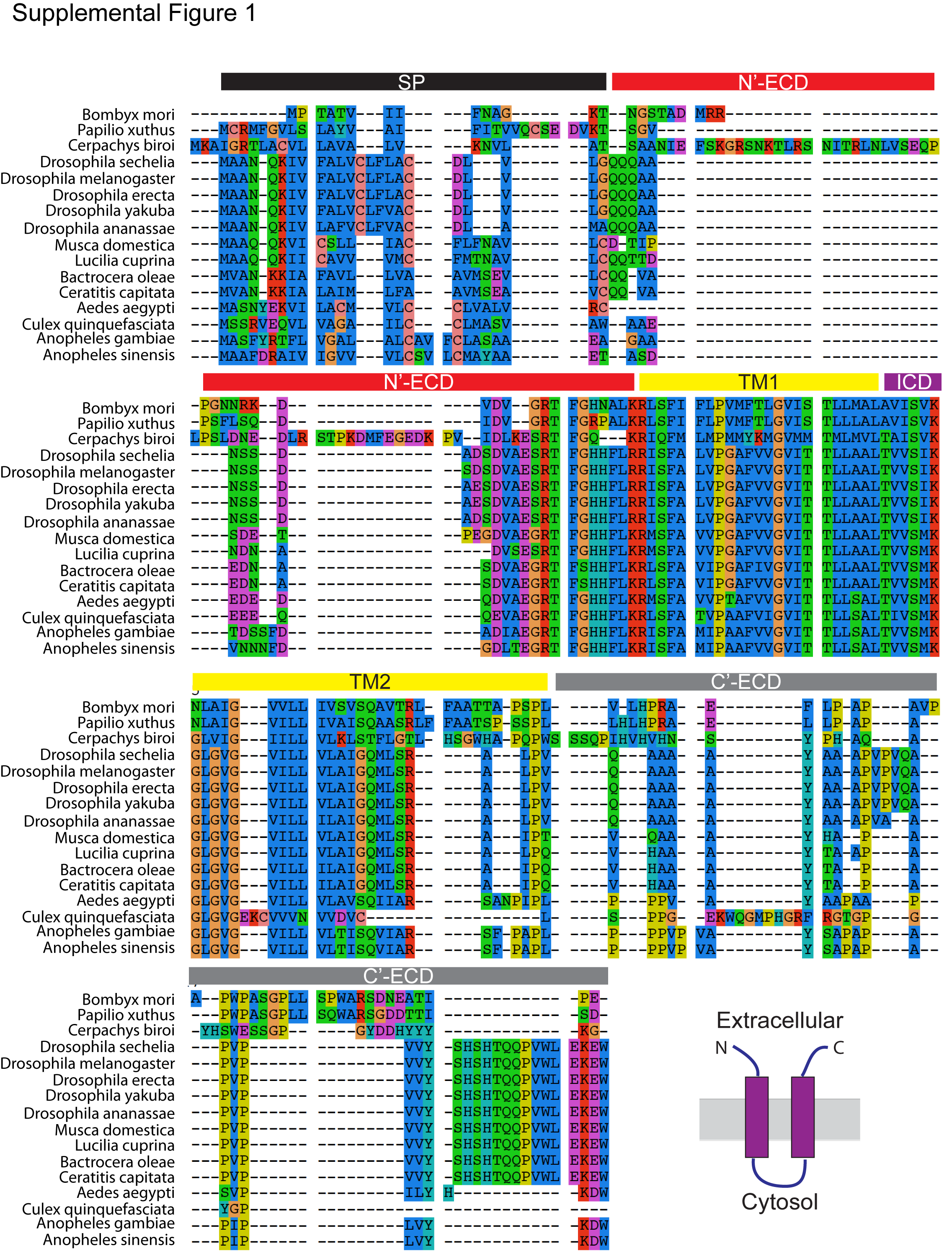

Supplement: S1 Fig — Prank software of the homologous sequences within the insect order was used. Colored bars indicate the protein domains. SP: Signal Peptide, N’-ECD: N-terminal Extracellular Domain, TM: Transmembrane domain, ICD: Intracellular Domain, C’-ECD: C-terminal Extracellular Domain. (TIF) [file pgen.1007852.s001.tif]

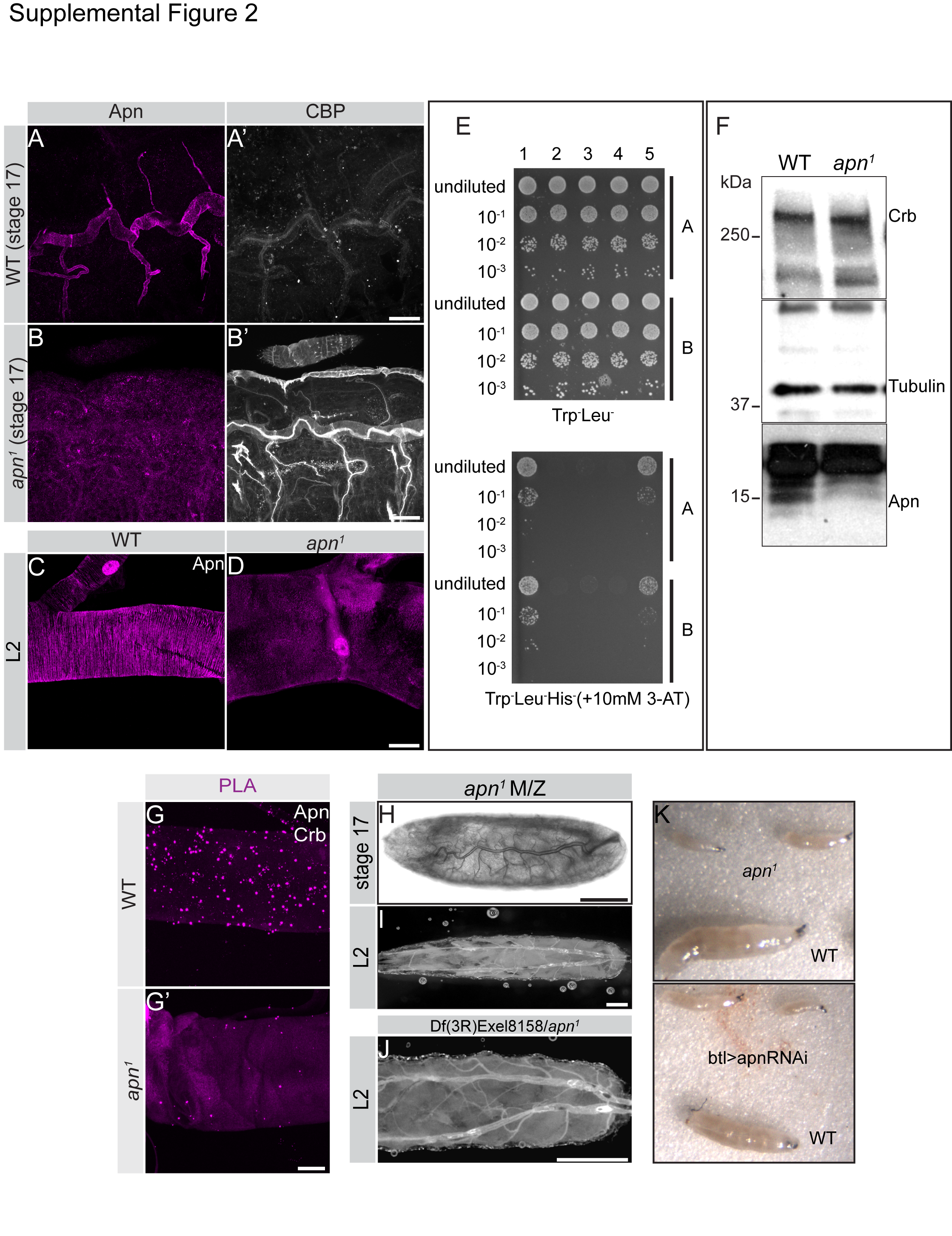

Supplement: S2 Fig — (A-D) Immunostaining of embryonic (A-B’) and larval (C, D) tracheae with anti-Apn antibody shows specific tracheal staining in wild type (WT) stage 17 embryos (A) and second instar (L2) larva (C). No Apn protein can be detected in apn1 mutant embryos (B) and second instar (L2) larva (D). Embryos/larvae in A’, B’ were additionally stained for chitin binding probe (CBP) to highlight the luminal matrix. Scale bars: 20μm. (E) Yeast-two-hybrid analysis detecting interaction between the bait (Crb) and the prey (Apn). The top plate shows growth on media lacking tryptophan and leucine, used to verify co-transformation of plasmids as well as a growth control. The bottom plate shows the same dilutions spotted on medium lacking additionally histidine and is used to confirm the interaction between bait and prey. Column 1 is the positive control whereas columns 2, 3, 4 represent the negative controls (2: pB102/pP55 empty vectors, 3: pB102 empty vector/CG15887, 4: Crb/pP55 empty vector), column 5 represents the interaction between Crb and Apn. For each interaction several dilutions (undiluted, 10−1, 10−2, 10−3) were spotted. Interactions were tested with two independent clones (A and B). (F) Western blot from lysates of larval tracheae showing the expression levels of Crb and Apn in WT- and apn1 mutants. Tubulin is used as loading control. The 15kDa, Apn-positive band is absent in the apn1 mutant extract. The higher molecular weight bands are unspecific. (G, G’) Proximity ligation assay (PLA) between WT and apn1 mutant larval tracheae using Apn and Crb antibodies shows that the interaction is abolished in mutants lacking apn as compared to wild type. Scale bar: 20μm. (H, I) apn1 mutant embryo (H) and larva (I) derived from germline clones (M/Z; maternal/zygotic). (H) No defects were observed in the tracheal tubes of mutant embryos. Scale bar: 100μm. (I) Defects appear at second larval instar with irregular and twisted tracheal tubes (I). Scale bar: 500μm. (J) Brightfield image of [file pgen.1007852.s002.tif]

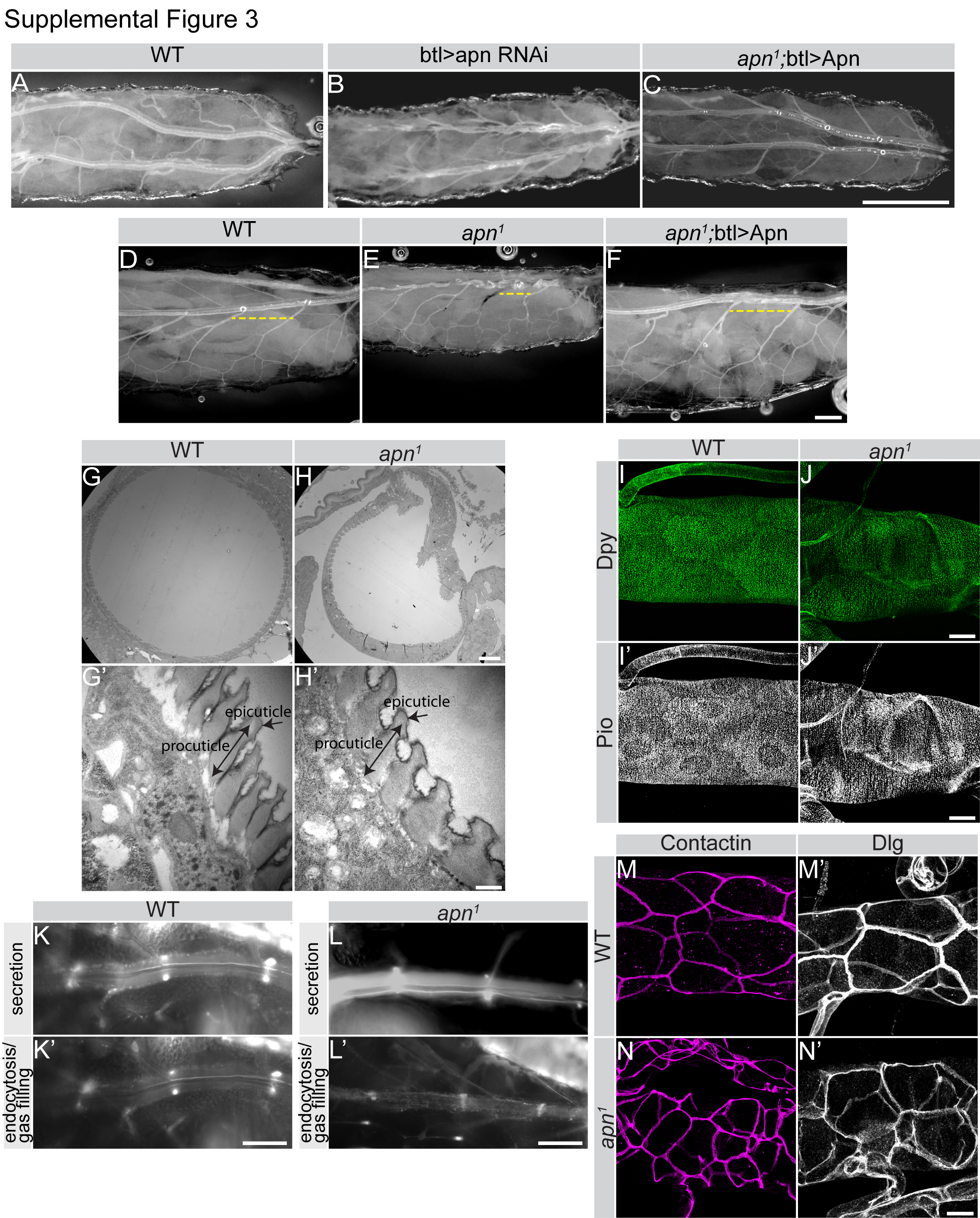

Supplement: S3 Fig — (A-C) Brightfield dorsal views of second instar larvae, showing the structure of tracheal tubes of wild type (WT) (A) and tracheal-specific apn down-regulation (btl>apn RNAi), which recapitulates the apn1 mutant tracheal defects (B). The tube morphology defects are partially rescued by tracheal expression of Apn (apn1; btl>Apn). Scale bar: 500μm. (D-F) The 9th metamer of the dorsal trunk (yellow dotted line) of apn1 mutant second instar larvae (E) is shorter than that of WT larvae (D). Tracheal expression of a transgene (apn1;btl>apn) rescues the metamer elongation defects of apn mutant larvae (F). Anterior is to the left. Scale bar: 200μm. (G-H’) Transmission electron micrographs of cross sections through a WT (G, G’) and apn1 mutant (H, H’) second instar trachea. (G–H) Axial views of the dorsal trunk (DT), G’ and H’ are higher magnifications to depict the larval cuticular ECM (epi- and procuticle) and the taenidial ridges. Scale bars: G, H 7.5μm; G’, H’ 700nm. (I-J’) Immunostaining of larval tracheal tubes with antibodies against the apical extracellular matrix (aECM) proteins Dumpy (Dp) (I, J) and Piopio (I’, J’). Scale bars: 20μm. (K-L’) Tracheal maturation of WT (K, K’) and apn1 mutant (L, L’) second instar larvae. Secretion of the luminal protein ANF-Cherry (E, F), as well as its clearance from the luminal space (K’, L’), are comparable between WT and apn1 mutants. Scale bars: 50μm. (M-N’) Immunostaining of WT and apn1 mutant tracheal tubes of second instar larvae with antibodies against the septate junction proteins Contactin (Cont) (M, N) and Discs Large (Dlg) (M’, N’). Scale bar: 20μm. (TIF) [file pgen.1007852.s003.tif]

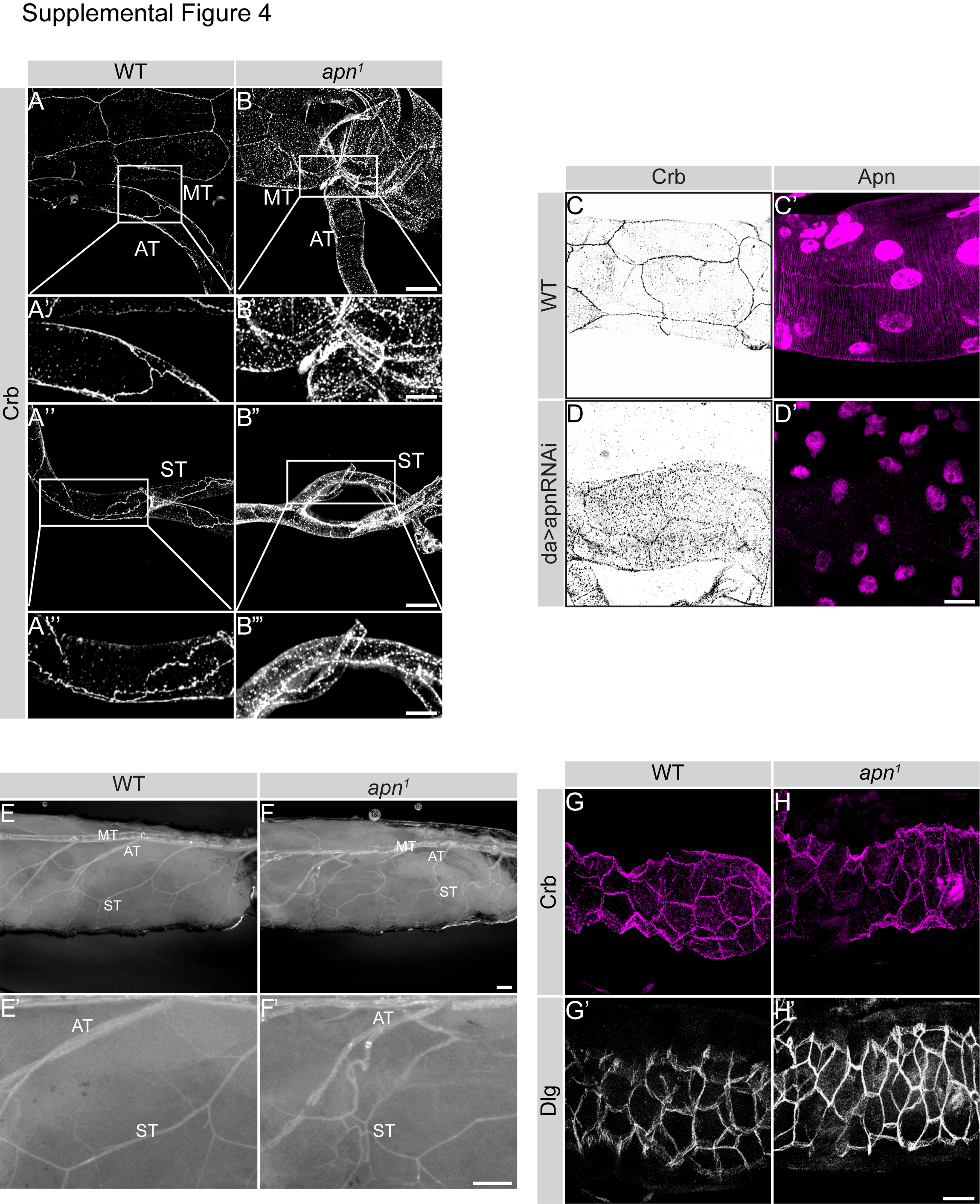

Supplement: S4 Fig — (A-B‴) Confocal projections showing tracheal tubes of wild type (WT, A-A’”) and apn1 mutant (B-B‴) second larval instar larvae, stained with anti-Crb. Crb localization is affected in multicellular tubes (MT), lateral branches [autocellular (AT) and seamless tubes (ST)] of mutant larvae. Scale bars: (A, B, A”, B”) 20μm and (A’, B’, A”‘, B”‘) 10μm. (C-D’) RNAi-mediated knockdown of apn by daughterless-Gal4 (da-Gal4) results in accumulation of Crb-positive cytoplasmic punctae (compare C and D) and strong reduction of Apn (compare C’ and D’). Nuclear Apn signal is considered to be unspecific. Scale bars: 20μm. (E-F’) Brightfield lateral views of second instar larvae showing the structure of multicellular tubes (MT), autocellular (AT) and seamless tubes (ST) of WT (E, E’) and apn1 mutants (F, F’). Scale bars: E, F 200μm; E’, F’ 1000μm. (G-H’) Confocal projections showing the salivary gland of WT (G, G’) and apn1 mutants (H, H’) second instar larvae, stained for Crb and Dlg. Scale bars: 20μm. (TIF) [file pgen.1007852.s004.tif]

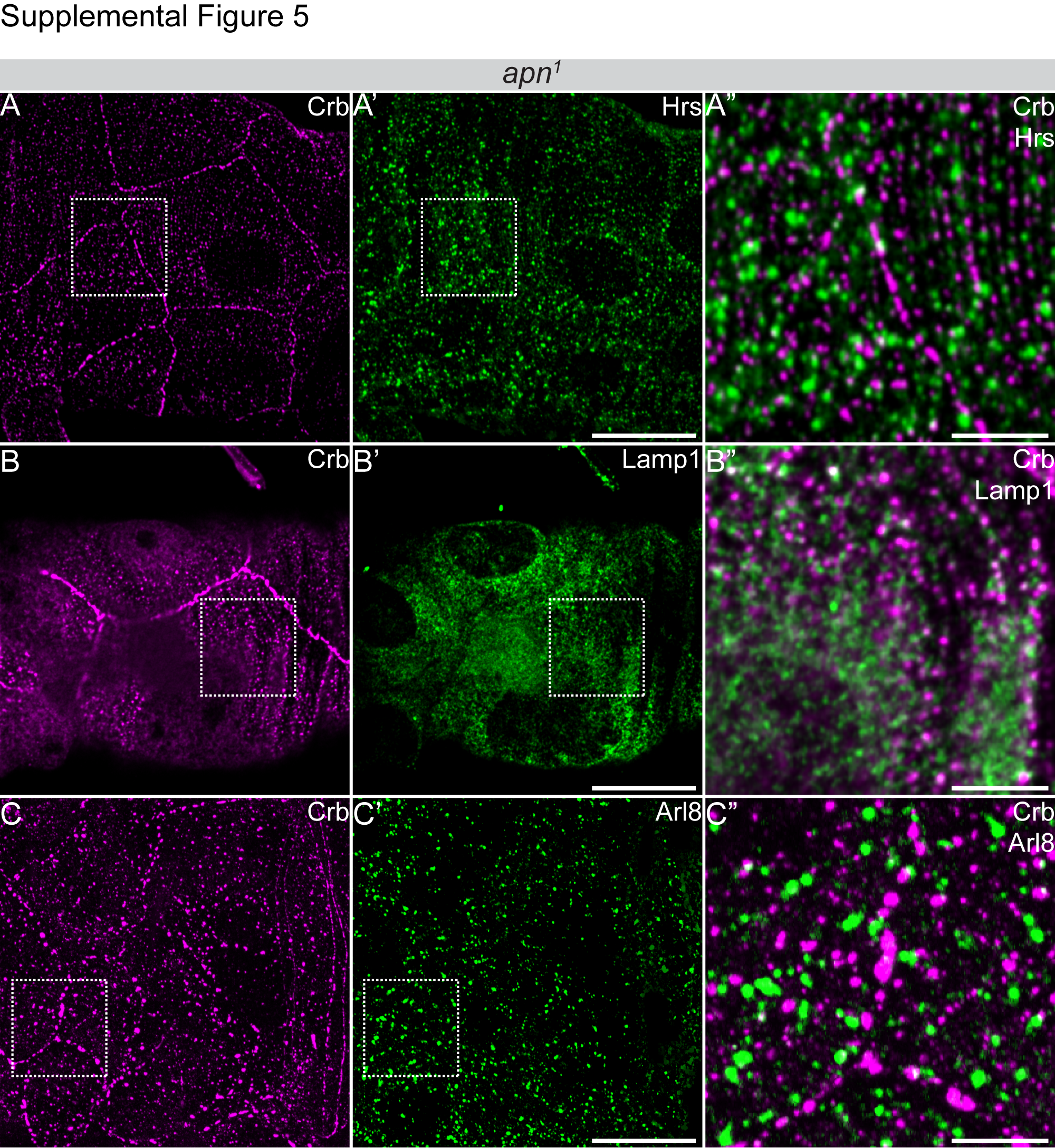

Supplement: S5 Fig — (A-A”) apn1 mutant tracheal tubes of second instar larvae immunostained for Crb (magenta) and Hrs (green). Magnification in A” shows hardly any co-localization of vesicular Crb and Hrs. (B-B”) apn1 mutant tracheal tubes of second instar larvae immunostained for Crb (magenta) and Lamp1 (green). Magnification in B” shows hardly any co-localization of vesicular Crb and Lamp1. (C-C”) apn1 mutant tracheal tubes of second instar larvae immunostained for Crb (magenta) and Arl8 (green). Magnification in C” shows hardly any co-localization of vesicular Crb and Arl8 staining. Scale bars: A, A’, B, B’, C, C’ 20μm; A”, B”, C” 5μm. (TIF) [file pgen.1007852.s005.tif]

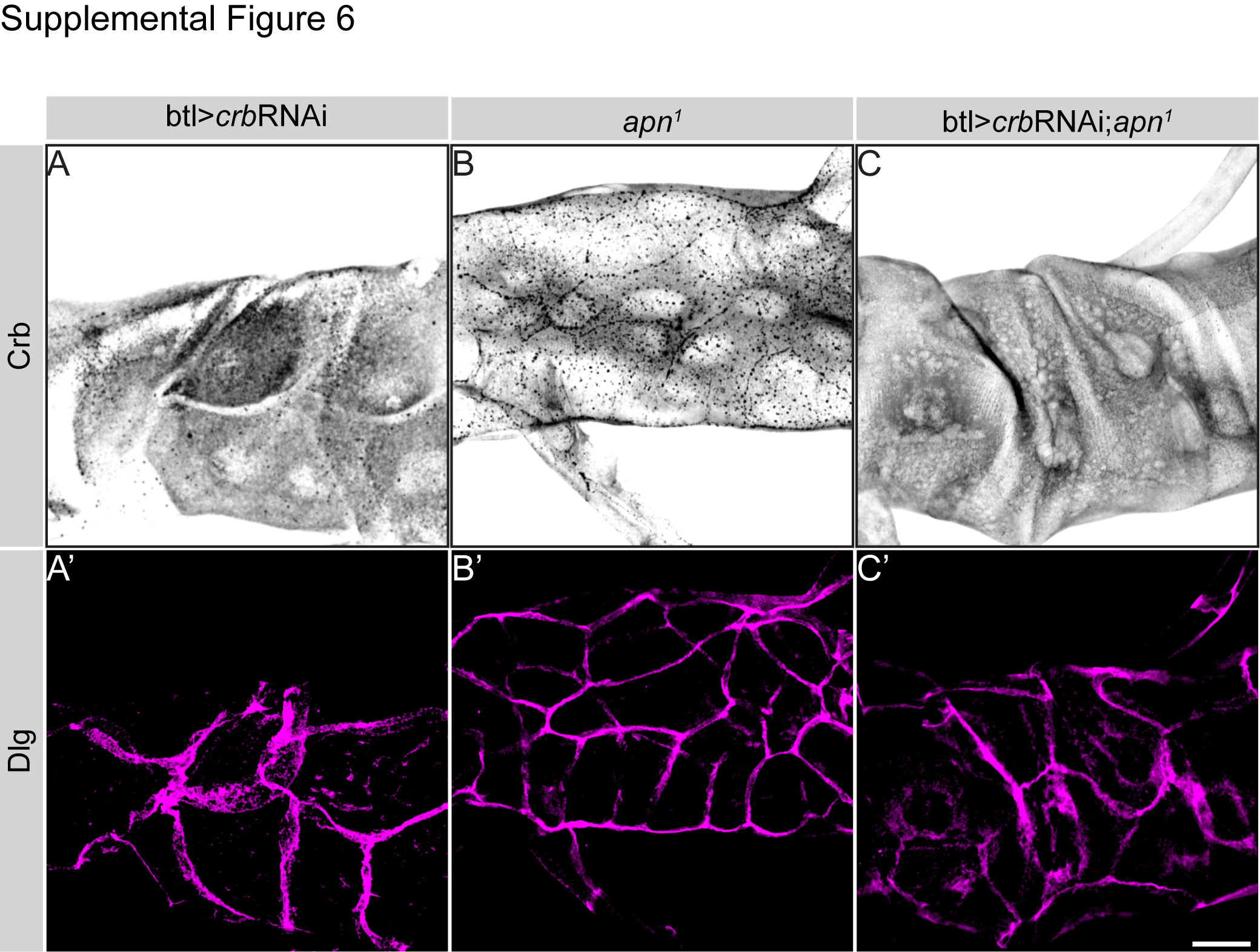

Supplement: S6 Fig — (A-C’) RNAi-mediated downregulation of Crb (A, C) results in depletion of Crb, but does not affect Dlg expression (A’, C’) in the dorsal trunk of second instar larvae. In apn1 mutants, Crb is detected in cytoplasmic punctae (B), whereas Dlg is properly localized baso-laterally in tracheal cells (B’). Projections of confocal sections of second instar tracheal tubes, stained for Crb (A-C), Dlg (green; A’-C’). Scale bars: 20μm. (TIF) [file pgen.1007852.s006.tif]
